# Temperature-Responsive Bottlebrush Polymers Deliver a Stress-Regulating Agent *In Vivo* for Prolonged Plant Heat Stress Mitigation

**DOI:** 10.1021/acssuschemeng.2c06461

**Published:** 2023-02-14

**Authors:** Yilin Zhang, Liye Fu, Michael R. Martinez, Hui Sun, Valeria Nava, Jiajun Yan, Kurt Ristroph, Saadyah E. Averick, Benedetto Marelli, Juan Pablo Giraldo, Krzysztof Matyjaszewski, Robert D. Tilton, Gregory V. Lowry

**Affiliations:** ^†^Department of Civil and Environmental Engineering, ^‡^Center for Environmental Implications of Nano Technology (CEINT), ^§^Department of Chemistry, ^∥^Department of Chemical Engineering, ^⊥^Department of Biomedical Engineering, Carnegie Mellon University, Pittsburgh, Pennsylvania 15213, United States; # Neuroscience Institute, Allegheny Health Network, Allegheny General Hospital, Pittsburgh, Pennsylvania 15212, United States; ∇ Department of Botany and Plant Sciences, University of California, Riverside, California 92521, United States; ○ Department of Civil and Environmental Engineering, Massachusetts Institute of Technology, Cambridge, Massachusetts 02139, United States

**Keywords:** heat stress, climate change, temperature responsive, agrochemical delivery, sustainable agriculture

## Abstract

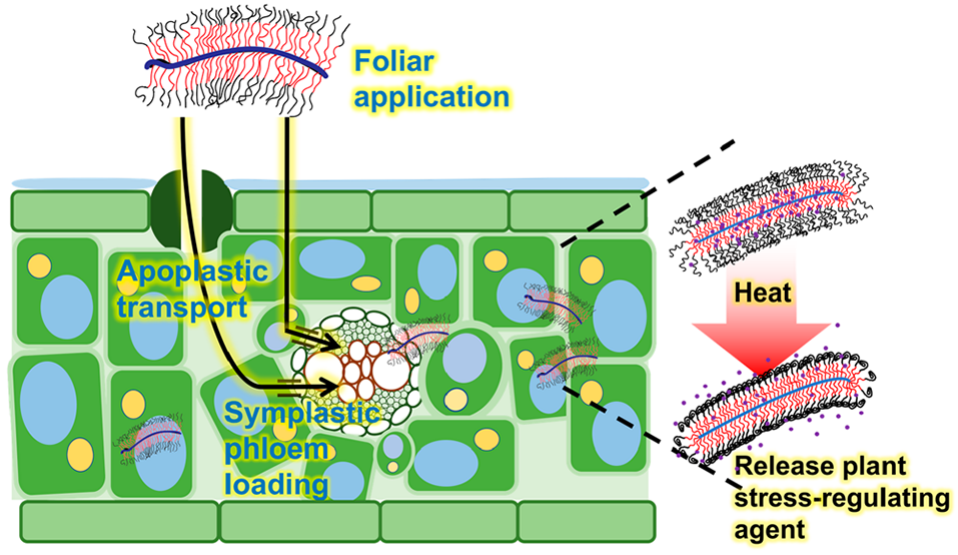

Anticipated increases in the frequency and intensity of extreme temperatures will damage crops. Methods that efficiently deliver stress-regulating agents to crops can mitigate these effects. Here, we describe high aspect ratio polymer bottlebrushes for temperature-controlled agent delivery in plants. The foliar-applied bottlebrush polymers had near complete uptake into the leaf and resided in both the apoplastic regions of the leaf mesophyll and in cells surrounding the vasculature. Elevated temperature enhanced the *in vivo* release of spermidine (a stress-regulating agent) from the bottlebrushes, promoting tomato plant (*Solanum lycopersicum*) photosynthesis under heat and light stress. The bottlebrushes continued to provide protection against heat stress for at least 15 days after foliar application, whereas free spermidine did not. About 30% of the ∼80 nm short and ∼300 nm long bottlebrushes entered the phloem and moved to other plant organs, enabling heat-activated release of plant protection agents in phloem. These results indicate the ability of the polymer bottlebrushes to release encapsulated stress relief agents when triggered by heat to provide long-term protection to plants and the potential to manage plant phloem pathogens. Overall, this temperature-responsive delivery platform provides a new tool for protecting plants against climate-induced damage and yield loss.

## Abstract 2

High aspect ratio temperature-responsive polymer bottlebrushes taken up by plant leaves can deliver stress-regulating agents into mesophyll cells to promote photosynthesis under heat stress for up to 15 days after foliar application, providing a large time window of protection against climate stress and promoting sustainability of agriculture.

## Introduction

Climate change-induced global warming is causing more frequent and severe heat stress incidents in crop plants that threaten global crop production.^1−6^ Plant heat stress leads to functional uncoupling of metabolic pathways that damage plant cell organelles,^7^ inhibit plant photosynthesis, and increase plant vulnerability to pathogens.^2,8^ This vulnerability lowers crop yields and profitability. For example, a 1 °C warmer climate in West Africa in 2000–2009 resulted in a 5%–20% reduction in crop yield,^9^ and a 3 °C global warming is predicted to result in $136 billion losses globally from decreased crop yields.^10^ It is anticipated that heat stress from higher maximum daily temperatures will decrease the productivity of major crops by up to 40% by 2070,^6^ while global food demand is predicted to increase by 60% before 2050.^11−15^ New materials and strategies are needed to improve sustainability of agriculture,^16−19^ make agriculture more resilient to heat stress, and meet the United Nations Sustainable Development Goals (Zero Hunger, Clean Water and Sanitation).^20^

There are a few potential ways to improve plant resilience to heat stress. Plant bioengineering may generate transgenic plants with heat stress tolerance.^21,22^ However, the challenges in understanding the physiological effects of the transgenes at the whole plant level,^23^ the high upstream production costs from plant regeneration and elite line propagation, and public concerns regarding biosafety of transgene products limit their application.^22^ Foliar application of active agents is another potential solution.^24−26^ For example, anionic cerium oxide nanoparticles (NPs) infiltrated into *Arabidopsis thaliana* leaves scavenged reactive oxygen species (ROS) in plants and alleviated heat stress.^8^ Foliar-applied ROS responsive star polymers scavenged ROS and delivered magnesium to chloroplasts, which improved plant stress tolerance.^27^ Plant stress-regulating agents such as glycine betaine, spermidine, and salicylic acid can also mitigate plant heat stress after foliar application^28−30^ but only provide short-term benefits and need to be applied during the stress event which can be difficult to predict precisely.^8,31^ Longer-term stress tolerance would require frequent and continuous active agent application, which generates more waste and increases application costs.^2^ Moreover, all of these studies only demonstrated agent delivery into treated leaves. Agent delivery into the plant vasculature and roots can also protect plants from pathogens when the plants are more vulnerable to diseases during heat stress.^2^ Existing approaches also cannot deliver the agent in response to the environmental stressors itself, e.g., elevated temperature for heat stress, which limits their abilities to provide immediate protection to plants when the stressors manifest. New approaches that inoculate the plants to confer heat stress tolerance by responding to elevated temperature to deliver stress-regulating agents to different plant organs will provide a new tool for managing heat stress in crops.

High aspect ratio nanomaterials can be promising tools for agent delivery in plants. The well-known tobacco mosaic virus (TMV), with an ∼20 nm diameter and ∼300 nm length, can infect plants after entry and transport efficiently inside of plants.^32^ Polyethylenimine-functionalized carbon nanotubes with ∼10–20 nm diameters and lengths up to ∼600 nm can be infiltrated into leaves to deliver genetic material into mature plants for transcription in leaf mesophyll cells.^33^ A chitosan-complexed single-walled carbon nanotube carrier selectively delivered and expressed plasmid DNA in chloroplasts.^34^ Although high aspect ratio nanomaterials have shown desired functionalities for agent delivery in plants, a high aspect ratio nanocarrier that can respond to heat and deliver agents into different plant organs to combat heat stress is not yet available.

In this study, we synthesized a temperature-responsive high aspect ratio poly[2-(2-bromoisobutyryloxy)-ethyl methacrylate-*graft*-poly(acrylic acid)-*block*-poly(*N*-isopropyl acrylamide)] P[BiBEM-*g*-(PAA-*b*-PNIPAm)] bottlebrush polymer that can inoculate plants to confer resistance to heat stress for extended periods of time by releasing the heat stress-regulating agents inside of the plants in response to elevated temperatures. The bottlebrush copolymers were loaded with spermidine (Spd), a plant stress-regulating agent, and they provided temperature-activated Spd release *in vivo* to manage plant heat stress for at least 15 days after foliar application. Bottlebrush copolymers with ∼10 nm diameters and lengths up to ∼300 nm were synthesized, and their uptake, phloem loading, and translocation in plants after foliar application were determined.

## Materials and Methods

### Materials

N-Isopropylacrylamide (NIPAm, 97%), *tert-*butyl acrylate (*t*BA, 98%), β-cyclodextrin (β-CD), 2-bromoisobutyryl bromide (BiBB, 98%), 1-methyl-2-pyrrolidone (NMP), dichloromethane (DCM), copper powder (Cu^0^, 99.7%, 45 cm^2^ g^–1^), crystal violet (CV, ≥90.0%), ethyl 2-bromoisobutyrate (EBiB, 98%), copper(I) bromide (CuBr, ≥99.995%), copper(II) bromide (CuBr_2_, ≥99.995%), potassium fluoride (KF, 99%), trifluoroacetic acid (TFA, ≥98%), spermidine (99%), basic alumina, and chloroform-d (CDCl_3_) were obtained from Sigma-Aldrich. The (2-trimetylsiloxy)ethyl methacrylate (HEMA-TMS) was purchased from Scientific Polymer Products. The tris(2-dimethylaminoethyl)amine (Me_6_TREN, ≥99%), gadolinium(III) chloride hexahydrate (GdCl_3_·6H_2_O, 99%), anisole (99%), and N,N-dimethylformamide (DMF, 99%) were purchased from Alfa Aesar. HNO_3_ (70%, trace metal grade) and H_2_O_2_ (30%, ACS grade) were purchased from Fisher Scientific. Dialysis bags with desired molecular weight cutoffs were purchased from Spectrum Lab (Spectra/Por 7). The *t*BA monomer was purified by passing through basic alumina to remove inhibitors. Other chemicals were used as received without further purification.

### Synthesis of P[BiBEM-*g*-(PAA-*b*-PNIPAm)] Bottlebrushes by Grafting

#### Synthesis of P[BiBEM-*g*-P*t*BA_50_]_320_ Polymer Bottlebrush

The polymer bottlebrushes were synthesized by a “grafting from” method. Syntheses of the PBiBEM_320_ and PBiBEM_1600_ polymer backbone followed our previous study.^35^ The P*t*BA block of the polymer bottlebrush was synthesized by normal ATRP.^36^ Briefly, 0.05 g (1 equiv) of PBiBEM_320_ initiator, 3.42 mL of *t*BA (52667 equiv), 6.27 mg of CuBr_2_ (63.2 equiv), 0.025 mL of Me_6_TREN (200 equiv), 2.74 mL of DMF, and 10.95 mL of anisole were mixed and sealed in a 25 mL Schlenk flask with a stir bar. The flask was deoxygenated by purging the reaction mixture with N_2_ for 40 min. The reaction mixture was then frozen by plunging it into liquid nitrogen. The flask was opened briefly to add 8.1 mg of CuBr (128 equiv) to the frozen reaction. The flask was sealed again and purged in liquid nitrogen for another 20 min to remove any air in the head space. The reaction was run at room temperature, and the monomer conversion was monitored by ^1^H NMR in CDCl_3_. The reaction was stopped at ∼30% conversion to yield P[BiBEM-*g*-P*t*BA_50_]_320_ polymer bottlebrushes. The product was dialyzed against methanol for three cycles (MWCO = 8000) to remove excess reagents. The molecular weight of polymer bottlebrushes was characterized with GPC-MALLS (Figure S2, Table S1).

#### Synthesis of P[BiBEM-*g*-(P*t*BA_50_-*b*-PNIPAm_50_)]_320_ Polymer Bottlebrush

The PNIPAm chain extension procedure was adopted from a previous study with some modifications.^2^ Briefly, 0.1 g of P[BiBEM-*g*-P*t*BA_50_]_320_ polymer bottlebrush (1 equiv), 0.42 g of NIPAm (79000 equiv), 0.67 mg of CuBr_2_ (63.2 equiv), 0.0025 mL of Me_6_TREN (190 equiv), 0.19 g of NaBr, and 18.65 mL of DMF were mixed and sealed in a 50 mL Schlenk flask with a stir bar. The flask was deoxygenated by purging the reaction mixture with N_2_ for 60 min. The reaction mixture was then frozen by liquid nitrogen. The flask was opened briefly to add 0.056 g of Cu^0^ powder (0.136 cm^–1^), sealed again, and purged in liquid nitrogen for another 30 min. The reaction was allowed to warm to room temperature, and the monomer conversion was monitored by ^1^H NMR in CDCl_3_. The reaction was stopped at ∼20% conversion to yield P[BiBEM-*g*-(P*t*BA_50_-*b*-PNIPAm_50_)]_320_ polymer bottlebrush. The product was purified by dialysis against methanol for three cycles (MWCO = 8000). The chemical composition of the product was verified by ^1^H NMR in CDCl_3_ (Figure S1c). Synthesis procedures of bottlebrush polymers with other arm compositions and backbone lengths are reported in the Supporting Information.

#### Hydrolysis of P[BiBEM-*g*-(P*t*BA-*b*-PNIPAm)] Bottlebrushes

A 0.5 g mass of synthesized polymer was dissolved in 10 mL of DCM with magnetic stirring. The polymer solution was then placed in an ice bath, and 1 mL of TFA was added into the star polymer solution. The reaction mixture was sealed and allowed to warm to room temperature and to react for ∼12 h. The resulting solution was dialyzed against methanol for three cycles (MWCO = 8000) to remove excess TFA.

#### Atomic Force Microscopy

Atomic force micrographs were obtained using a Cypher VRS AFM (Asylum Research). All samples were diluted to 10 mg L^–1^ for AFM imaging, in order to have an optimal density of features on the substrate. In a typical experiment, a 10 μL aliquot of diluted sample was dropped on a freshly cleaved mica surface (ϕ = 10 mm, Ted Pella) and air-dried before imaging. Images were acquired by tapping mode in air, at a scan rate of 4–8 Hz and a resolution of 256 × 256 pixels per image, using FS1500AuD (Asylum Research) probes.^37^

#### CV and Spd Loading and *In Vitro* Release from P[BiBEM-*g*-(P*t*BA-*b*-PNIPAm)] Bottlebrushes

To load CV into the star polymers, 10 mg of P[BiBEM-*g*-(P*t*BA-*b*-PNIPAm)] bottlebrushes was first dissolved into 5 mL of a 0.05 M NaOH aqueous solution. The sample was sonicated in an ice bath for 30 min (iSonic P4800, 60 W). The pH of the polymer solution was adjusted to 6.5 by 0.1 M NaOH or 0.1 M HCl aqueous solution. Then, 20 mg of CV or Spd was added into the star polymer solution. The mixture was vortex mixed for 1 day. The resulting solution was dialyzed against 2 L of Milli-Q water (MWCO = 8000) for two cycles to remove free CV or Spd. The CV concentration in the dialysate was measured by UV–vis spectrophotometry (Agilent Cary 4000) at a 590 nm absorbance wavelength, and the Spd concentration was assessed with Zincon dye according to a previously published protocol.^38^ CV and Spd loadings in the polymer bottlebrushes were calculated from the mass balance.

To assess the temperature and pH-responsive release properties of the CV-loaded polymer bottlebrushes, controlled release experiments were conducted at either 20 or 40 °C in 10 mM phosphate buffer at pH 4.5 by adjusting the pH of a 10 mM NaH_2_PO_4_ solution to 4.5 with 0.1 M HCl and NaOH, at pH 7.5 by mixing 1.91 mM NaH_2_PO_4_ and 8.09 mM Na_2_HPO_4_. In the typical procedure, 4 mL of a CV-loaded polymer bottlebrush solution was dialyzed against 100 mL of phosphate buffer solution (MWCO = 8000). The dialysate was sampled and measured for CV concentration by UV–vis spectrophotometry at multiple time points to assess the agent release profiles of each polymer bottlebrush under the different temperature and pH conditions.

The Spd release was examined in 10 mM acetate buffer at pH 4.5 (5.5 mM acetic acid, 4.5 mM sodium acetate) or 7.5 (10 mM sodium acetate). In the typical procedure, 4 mL of Spd-loaded bottlebrush solution was dialyzed against 100 mL of acetate buffer solution (MWCO = 8000). For each measurement, 0.6 mL of dialysate was mixed with 0.6 mL of Milli Q water, 0.15 mL of 1 M acetate buffer at pH 4, and 0.15 mL of 0.4 mM Zincon stock solution and shaken for 40 s before analysis by absorbance at 599 nm with UV–vis. The dialysate was sampled and measured at multiple time points to acquire Spd release profiles.

The *in vitro* CV release was also assessed in simulated phloem prepared according to previous studies (Table S2).^39−41^ The pH of simulated phloem was 7.0. The controlled release experiments were conducted at either 20 or 40 °C in simulated phloem. The dialysate was sampled and measured for CV concentration by UV–vis spectrophotometry at multiple time points to assess the agent release profiles of each polymer bottlebrush under different temperatures.

#### Plant Growth

For plant photosynthesis, hyperspectral imaging, and polymer nanocarrier uptake and transport studies, tomato (*Solanum lycopersicum*) seeds were rinsed by Milli-Q water two times (50 seeds in 2× 50 mL water) before being surface sterilized with 10% (v/v) bleach for 3 min, then thoroughly rinsed by Milli-Q water five times (5× 50 mL water). The sterilized seeds were germinated in a Petri dish on water-soaked filter paper in the dark for 10 days. The germinated seedlings were then transplanted to 100 mL plastic specimen cups (Vakly). Each seedling was grown hydroponically using 1/4 strength Hoagland’s solution aerated using air pumps. The plants were grown at room temperature (∼20 °C) with a 16 h light and 8 h dark cycle. The plants were used for foliar uptake and photosynthesis experiments after 30 days of growth.

#### Tracking Polymer Bottlebrush Distribution in Exposed Leaves by Enhanced Dark Field Hyperspectral Imaging (DF-HSI)

Five drops of 10 μL each (50 μL total) of the CV-loaded polymer suspension was applied onto the adaxial surfaces of tomato leaves by drop deposition. The spreading agent Silwet L-77 was added into the polymer solution at 0.1 vol % before foliar application. Plant leaf cross sections were prepared by a Leica CM1950 cryotome. The tomato leaves were frozen in Neg-50 frozen section medium (Thermo Scientific) with liquid nitrogen before being cut into 50 μm thick sections.

The distributions of CV-loaded bottlebrushes in plant leaf mesophyll and cross sections were studied by enhanced dark-field hyperspectral imaging (DF-HSI). This enhanced resolution dark-field microscope system (BX51, Olympus, USA) was equipped with a 150 W halogen light source (Fiber-Lite, Dolan-Jenner, USA) and a hyperspectral camera (CytoViva hyperspectral imaging system 1.4). The leaves were observed in oil immersion at 60× magnification. Hyperspectral images were acquired using 75% light source intensity and 0.1–0.25 s acquisition per line and corrected for the lamp contribution. The hyperspectral libraries were built using images of leaves exposed to the different CV-loaded star polymers and polymer bottlebrushes (Figure S4).^2^ All contributions of the spectrum contained in control images were background subtracted from exposed samples before image analysis. The hyperspectral libraries were used to map the locations of CV-loaded polymers in hyperspectral images of dosed leaves. A spectral angular mapping algorithm (SAM, ENVI 5.2) was used to identify the pixels matching the loaded polymer hyperspectral libraries (angles ≤0.085 rad were considered similar) on bands 1–177 (between 400 and 670 nm). Each pixel identified that way was highlighted in red. All the hyperspectral images were acquired at cross-section focus. Because of the narrow depth of field (less than a μm), signals of CV-loaded polymers were only mapped by SAM in the focal plane shown in the pictures, and out-of-focus CV-loaded star polymers and bottlebrushes adsorbed on top or under the focus plane were not mapped, in agreement with previous studies, which allows distinguishing polymers inside vs outside cells.

#### Star Polymer and Bottlebrush Foliar Exposure, Uptake, and Transport in Tomato Plants

The Gd^3+^ loading into star polymers and polymer bottlebrushes followed our previously published procedure.^2^ The Gd loading results are shown in Table S3. A 0.1 vol % Silwet L-77 spreading agent was added to Gd-loaded star polymer and bottlebrush solutions. Each polymer treatment was examined with five plants (five biological replicates). Four droplets of a 5 μL Gd-polymer solution at either 1g L^–1^ mass concentration or 9 × 10^16^ polymers L^–1^ number concentration were applied to the second true leaf of each of the five tomato plants per treatment. The plants were harvested 3 days after exposure. The plants were cut into five parts: the leaf where the Gd-loaded polymer solutions were applied (denoted as “exposed zone”), leaves at growth stages higher than exposed leaves (denoted as “younger leaf”), leaves at growth stages lower than exposed leaves (denoted as “older leaf”), main stem of the entire plant (denoted as “stem”), and roots (denoted as “root”). All plant samples were dried in an oven at 105 °C for 48 h to fully remove water from the tissue. The dried plants were digested with a 2:1 v/v mixture of 1 mL concentrated HNO_3_ and a 30% H_2_O_2_ aqueous solution heated to 100 °C for 45 min (protocol adapted from EPA Method 3050b^42^). Post digestion, all samples were diluted to 5% HNO_3_ by Milli-Q water and filtered by a 0.45 μm PTFE syringe filter before analysis by ICP-MS (Agilent 7700X).

#### Heat and Light Stress

In a photosynthesis assessment study, four droplets of a 5 μL Spd-loaded P[BiBEM-*g*-(PAA_50_-*b*-PNIPAm_50_)]_320_ polymer bottlebrush (0.5 g L^–1^ polymer concentration with 0.18 g L^–1^ loaded Spd) were applied to adaxial surfaces of tomato leaves with 0.1 vol % Silwet L-77. Then, 0.5 g L^–1^ of unloaded bottlebrush, 0.18 g L^–1^ of free Spd, and Milli-Q water (control) were also applied to tomato leaves with 0.1 vol % Silwet L-77 using the same approach. The bottlebrushes were allowed to interact with plant mesophyll cells for 24 h. Photosynthesis measurements were performed on the polymer treated leaves.

The carbon (carbon assimilation rate versus intercellular CO_2_ concentration, A-Ci) and light response (carbon assimilation rate versus photosynthetic active radiation, A-PAR) curves of treated leaves were measured 24 h after treatments before stress conditions. The gas chamber of Li-Cor was used to create a simultaneous heat and light stress conditions (T = 40 °C, 2000 μmol m^–2^ s^–1^ PAR, RH = 40%) for 1.5 h. The carbon and light response curves were measured again and compared with the curves acquired before stress. A-Ci curves were performed at 1200, 1000, 800, 600, 400, 200, 100, 50, and 0 ppm of Ci at 40 °C under 2000 μmol m^–2^ s^–1^ PAR light. The A-PAR curves were acquired at 1200, 900, 600, 400, 300, 200, 100, 50, and 0 μmol m^–2^ s^–1^ PAR at 40 °C, 400 ppm of Ci. Light-adapted (PhiPSII) chlorophyll fluorescent tests were also performed before and after stress conditions. The A-Ci curves were analyzed by fitting A and *C*_c_ to extract *V*_Cmax_ according to a previously reported model for C_3_ plants:^43^
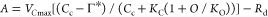
1where *V_C_*_max_ is the maximum carboxylation rate, *C*_c_ is the CO_2_ partial pressure in Rubisco, Γ* is the photorespiratory compensation point, *O* is the partial pressure of oxygen, *R*_d_ is the mitochondrial respiration rate, *K*_C_ and *K*_O_ are Michaelis constants of Rubisco for carbon dioxide and oxygen, respectively. The quantum yield of CO_2_ assimilation (PhiCO_2_) was acquired by calculating the slopes of A-PAR curves at 200, 100, 50, and 0 μmol m^–2^ s^–1^ PAR.^8^

## Results and Discussion

### Synthesis and Characterization of P[BiBEM-*g*-(PAA-*b*-PNIPAm)] Polymer Bottlebrushes with Different Aspect Ratios

**Figure 1:**
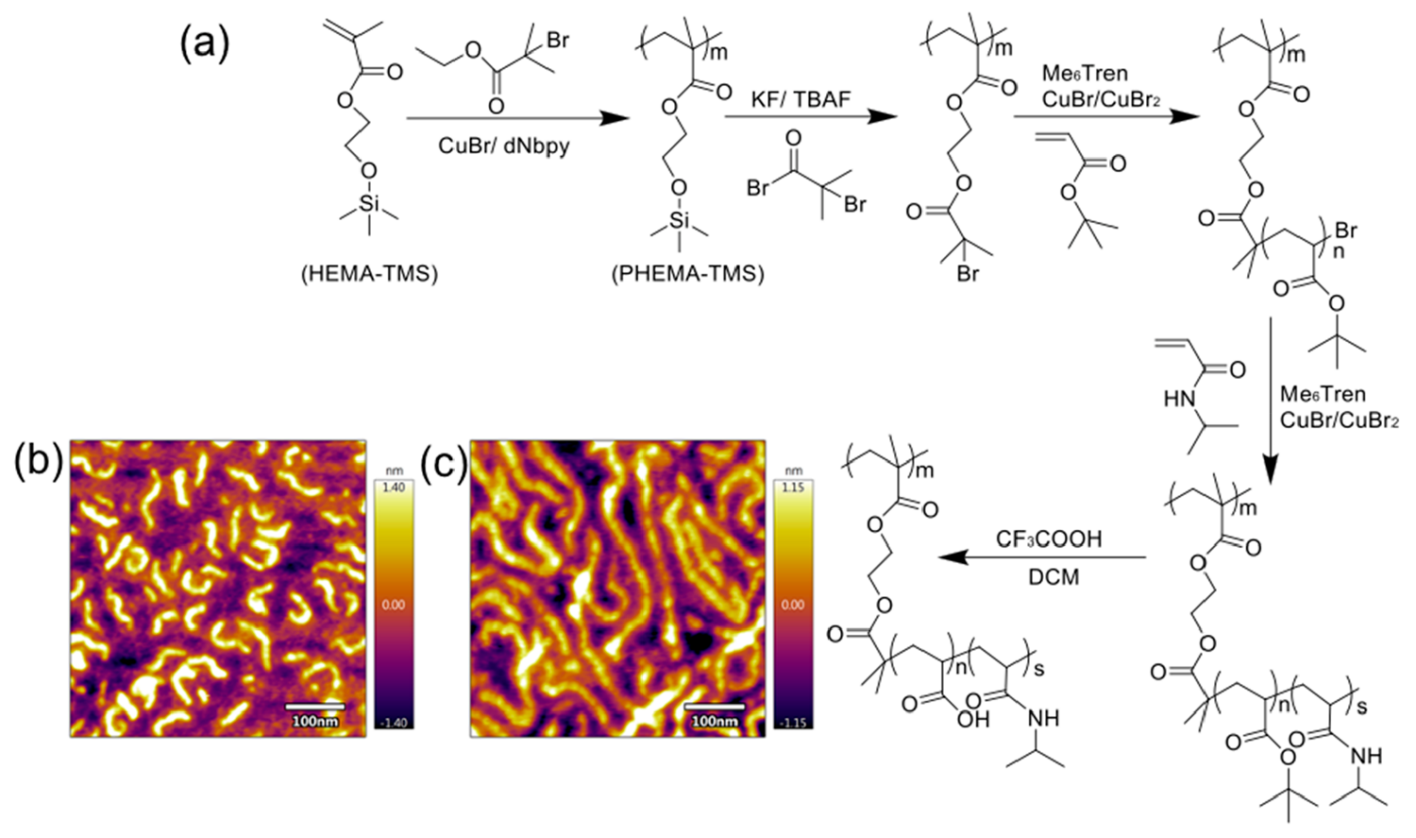
(a) Synthesis steps of the temperature-responsive high aspect ratio P[BiBEM-*g*-(PAA-*b*-PNIPAm)] polymer bottlebrushes. Atomic force microscope height images of (b) P[BiBEM-*g*-(PAA_50_-*b*-PNIPAm_50_)]_320_ (SBB50) and (c) P[BiBEM-*g*-(PAA_50_-*b*-PNIPAm_50_)]_1600_ (LBB50) polymer bottlebrushes.

To provide polymer nanocarriers with a range of temperature-responsive slow-release profiles and different aspect ratios, polymer bottlebrushes with 320 or 1600 poly(acrylic acid)-*block*-poly(*N*-isopropyl acrylamide) (PAA-*b*-PNIPAm) block copolymer arms and two different PAA to PNIPAm ratios were synthesized by a grafting approach as shown in Figure 1a.^35,36^ The chemical compositions of polymer bottlebrushes were confirmed by proton nuclear magnetic resonance (^1^H NMR) and gel permeation chromatography (GPC) (Figures S1 and S2, Table S1). The theoretical molecular weights of polymer bottlebrushes were calculated according to the molar ratio between P*t*BA and PNIPAm from their ^1^H NMR spectra and molecular weights of P[BiBEM-*g*-P*t*BA] bottlebrushes measured by GPC. The calculated theoretical molecular weights (*M*_n_) of P[BiBEM-*g*-(PAA_50_-*b*-PNIPAm_50_)]_320_ (denoted as SBB50), P[BiBEM-*g*-(PAA_50_-*b*-PNIPAm_150_)]_320_ (SBB150), P[BiBEM-*g*-(PAA_50_-*b*-PNIPAm_50_)]_1600_ (LBB50), and P[BiBEM-*g*-(PAA_50_-*b*-PNIPAm_150_)]_1600_ (LBB150) polymer bottlebrushes are shown in Table 1.

**Table 1:** Theoretical Number Average Molecular Weight (*M*_n_), Number Average Hydrodynamic Diameter (*D*_h_), Electrophoretic Mobility, and Apparent Zeta Potential (ζ) of Bottlebrush Polymers

Sample	*M*_n_^a^ (g mol^–1^)	*D*_h_ (nm)^b^	Electrophoresis mobility (μm cm V^–1^ s^–1^)^c^	ζ (mV)^d^
P[BiBEM-*g*-(PAA_50_-*b*-PNIPAm_50_)]_320_ (SBB50)	2.96 × 10^6^	39.5 ± 5.4	–1.29 ± 0.04	–16.5 ± 0.6
P[BiBEM-*g*-(PAA_50_-*b*-PNIPAm_150_)]_320_ (SBB150)	6.58 × 10^6^	41.8 ± 1.3	–0.82 ± 0.06	–10.5 ± 0.7
P[BiBEM-*g*-(PAA_50_-*b*-PNIPAm_50_)]_1600_ (LBB50)	1.53 × 10^7^	105.5 ± 5.3	–1.26 ± 0.05	–15.4 ± 0.6
P[BiBEM-*g*-(PAA_50_-*b*-PNIPAm_150_)]_1600_ (LBB150)	3.34 × 10^7^	91.5 ± 4.9	–0.71 ± 0.09	–9.1 ± 1.2

a Theoretical number average molecular weight calculated according to the chemical composition.

b Hydrodynamic diameters were determined in water at 100 mg L^–1^ of polymer concentration at pH 6.5 (10 mM NaCl) by dynamic light scattering (Malvern Zetasizer Nano ZS). Bottlebrush size distributions are shown in Figure S3.

c Electrophoretic mobility measured with 100 mg L^–1^ of polymer solution at pH 6.5 with 10 mM NaCl (Malvern Zetasizer Nano ZS).

d Apparent zeta potentials were calculated from the mobility using the Smoluchowski model.

Morphologies of the bottlebrushes were confirmed by atomic force microscopy. The bottlebrush polymers have worm-like structures, with lengths around 80 nm for the 320-armed SBB50 (Figure 1b) and around 300 nm for the 1600-armed LBB50 bottlebrushes (Figure 1c). The hydrodynamic diameters of the polymer bottlebrushes were directly proportional to the lengths of backbone, rather than the DPs of the arms. The SBB50 and SBB150 polymer bottlebrushes with different DPs in each arm but the same number of arms were both ∼40 nm (Table 1, Figure S3a, b) The average hydrodynamic diameters of 1600-armed LBB50 and LBB150 polymer bottlebrushes were both ∼100 nm (Table 1, Figure S3c, d). All of the bottlebrushes had a negative electrophoretic mobility and apparent zeta potential due to deprotonated carboxylic acid groups in their PAA cores. The bottlebrush polymers with longer PNIPAm outer blocks in the arms had less negative apparent zeta potentials (Table 1). This is consistent with the nonionic PNIPAm chains shifting the plane of shear further away from the negatively charged core.^44^

### Agent Loading and *In Vitro*-Controlled Release for Polymer Bottlebrushes with Different PAA to PNIPAm Ratios

**Figure 2:**
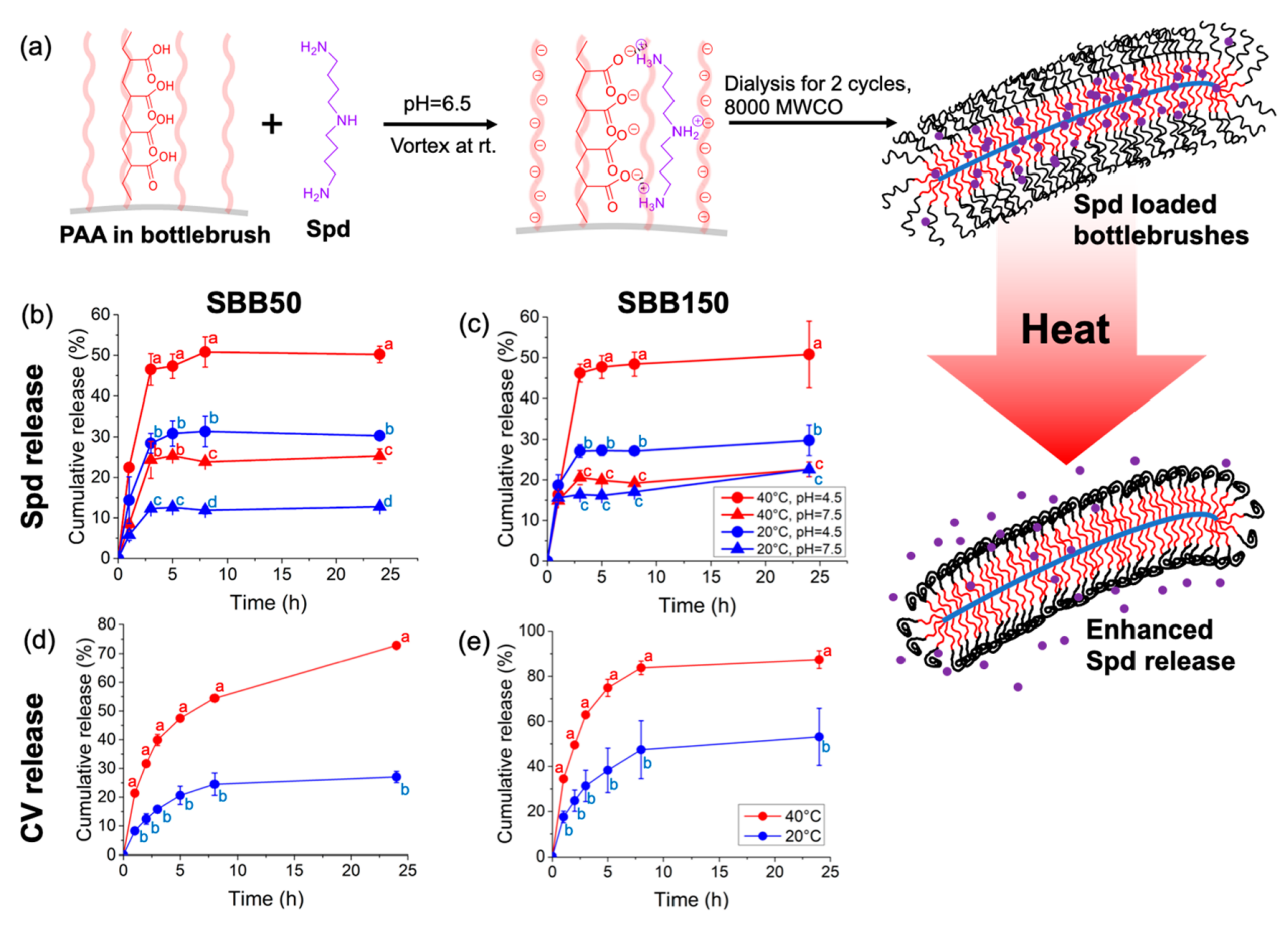
(a) Schematic showing the spermidine (Spd) loading into the polymer bottlebrushes and high temperature induced Spd release. Spd release profiles of (b) P[BiBEM-*g*-(PAA_50_-*b*-PNIPAm_50_)]_320_ (SBB50) and (c) P[BiBEM-*g*-(PAA_50_-*b*-PNIPAm_150_)]_320_ (SBB150) polymer bottlebrushes over 24 h at 20 and 40 °C in 10 mM acetate buffer adjusted to pH 4.5 or 7.5. CV release profiles of (d) P[BiBEM-*g*-(PAA_50_-*b*-PNIPAm_50_)]_320_ (SBB50) and (e) P[BiBEM-*g*-(PAA_50_-*b*-PNIPAm_150_)]_320_ (SBB150) polymer bottlebrushes over 24 h at 20 °C (blue) and 40 °C (red) in simulated phloem (Table S2). Note that Spd release in simulated phloem could not be measured due to analytical limitations. ANOVA test followed by Fisher’s least significant difference test for multiple comparisons, *P* ≤ 0.05. Error bars represent standard deviations from three replicates.

The polymers are intended to carry the positively charged active agent, spermidine (spd), into the plant, so we measured the amount of spermidine that could be loaded into each polymer morphology and the impact of spermidine loading on the properties of the polymers. Spd is a stress-regulating agent and plant growth regulator that can alleviate abiotic stress such as heat and salinity stresses after foliar application.^45−47^ Crystal violet (CV) is loaded into other polymers to visualize their distributions in the plant leaves. CV has also been used as a fungicide for crop protection, and the amine groups in CV are common in plant antimicrobial agents.^2,32^ The Spd loading procedure and reaction mechanism are shown in Figure 2a. Spd or CV are loaded into bottlebrushes at pH 6.5 through electrostatic interactions between negatively charged carboxylates in PAA and positively charged amine groups in Spd and CV. The Spd loading efficiency, expressed as loaded mass of Spd per unit mass of bottlebrushes for SBB50 and SBB150 bottlebrushes were 0.36 ± 0.07 g Spd g^–1^ and 0.12 ± 0.02 g Spd g^–1^, respectively. This corresponds to from 5500 ± 910 to 7300 ± 1400 Spd molecules bound to each bottlebrush (∼0.34–0.45 Spd per carboxylic group). The CV loadings were 0.63 ± 0.08 g CV g^–1^ for SBB50 and 0.27 ± 0.02 g CV g^–1^ for SBB150 bottlebrushes, corresponding to from 4400 ± 320 to 4600 ± 580 CV molecules bound to each bottlebrush (∼0.28–0.29 CV per carboxylic group). Spd and CV mass loading efficiencies are lower for bottlebrushes with higher PNIPAm content, while the numbers of CV and Spd loaded into each bottlebrush molecule were similar for both SBB50 and SBB150 bottlebrushes because they have the same number of PAA (∼16,000) residues. This indicates that the PNIPAm blocks do not interfere with loading efficiency.

The Spd- and CV-loaded polymer bottlebrushes were first tested *in vitro* to evaluate the effects of chain composition, pH, and solution compositions on the temperature-responsive agent release profiles of polymer bottlebrushes. The temperature-dependent Spd release profiles were acquired at plant-relevant pH values (4.5 and 7.5) in 10 mM acetate buffer. The pH in apoplastic space of plant tissue can reach ∼4.5 under stressed conditions, and the pH of plant chloroplast can reach ∼7.5 with chloroplast cytoplasmic pH of ∼7.0 and stroma pH of ∼8.0.^2,48^ Therefore, 4.5–7.5 is the relevant pH conditions in different plant organs and cell organelles. The SBB50 bottlebrush exhibited high temperature-enhanced Spd release at both pH 4.5 and 7.5, with higher overall Spd release at pH 4.5 (Figure 2b). The SBB150 bottlebrush only exhibited high temperature-enhanced Spd release at pH 4.5, but not 7.5 (Figure 2c). Spd has p*K*_a_ values ranging from 8 to 11, and the p*K*_a_ of PAA is around 4.5.^49^ Therefore, a pH change from 4.5 to 7.5 significantly affects the protonation state of PAA but not Spd. The protonation of PAA at pH 4.5, and thus the weaker attraction between Spd and PAA, potentially enabled higher Spd release and better temperature responsiveness from polymer bottlebrushes at pH 4.5. The SBB50 bottlebrush was used in the *in vivo* experiments due to its distinct temperature responsiveness at both high and low pH.

The temperature-responsive CV release profiles were acquired in simulated phloem at pH 7^41,50,51^ (see Table S2 for liquid composition) to assess the potential effectiveness of polymer bottlebrushes in delivering antimicrobial agents to target phloem pathogens during heat stress. As shown in Figure 2d and e, both the SBB50 and SBB150 bottlebrushes yielded higher cumulative CV releases at 40 °C than at 20 °C. A similar release behavior was observed in a 10 mM phosphate buffer (Figure S4), indicating the ingredients in phloem sap, including sucrose, amino acids, and metal cations, did not inhibit the temperature responsiveness of bottlebrushes. Additional details of temperature-responsive CV release in a phosphate buffer are provided in the SI.

### Spd Delivery with Polymer Bottlebrushes Promotes Plant Photosynthesis during Heat and Light Stress for at Least 15 Days after Application

Following the general trend of global warming, temperatures in the mid-to-high 40s °C are causing more frequent damage to crops and substantial losses.^52^ Heat and excess light stress can cause reactive oxygen species (ROS) accumulation in plant protoplast.^2,8,53^ Excess ROS damages components of the photosynthetic system in the chloroplast, including chlorophyll, lipids, chloroplast genomes, and photosystem II reaction center (PSII RC) proteins.^8,54,55^ Alleviating plant stress and managing ROS accumulation are necessary for crop plant protection. Foliar-applied Spd can improve plant stress tolerance by enhancing antioxidant enzyme activity and chlorophyll fluorescence.^29,56^ Only SBB50 bottlebrushes were tested given their higher agent loading capacity and sharper temperature response *in vitro*. The high Spd loading and enhanced Spd release at elevated temperatures suggest their potentials to regulate plant heat stress by delivering Spd into leaf mesophyll and releasing it during high temperature events when plants are under stress.

Spd-loaded SBB50 bottlebrushes, free Spd, unloaded SBB50 bottlebrushes, and a Milli-Q water (control) with 0.1 vol % Silwet L-77 were applied to mature tomato leaves to assess their impact on plant stress tolerance. Four droplets of 5 μL of a Spd-loaded SBB50 polymer bottlebrush (0.5 g L^–1^ of polymer concentration with 0.18 g L^–1^ of loaded Spd) were applied to each leaf. Then, 24 h after application, the plants were subjected to 1.5 h of heat and excess light exposure (40 °C and 2000 μmol m^–2^ s^–1^ photosynthetic active radiation, PAR). Several plant photosynthesis parameters were measured before and after stress, including the Rubisco carboxylation rate (*V*_*C*max_) and carbon assimilation rate versus the intercellular CO_2_ concentration (A-Ci) curve that quantifies the carbon reaction and the photosystem II quantum yield (PhiPSII), CO_2_ quantum yield (PhiCO_2_), and PSII variable fluorescence over maximal fluorescence (Fv/Fm) that quantify light reaction activity.

**Figure 3:**
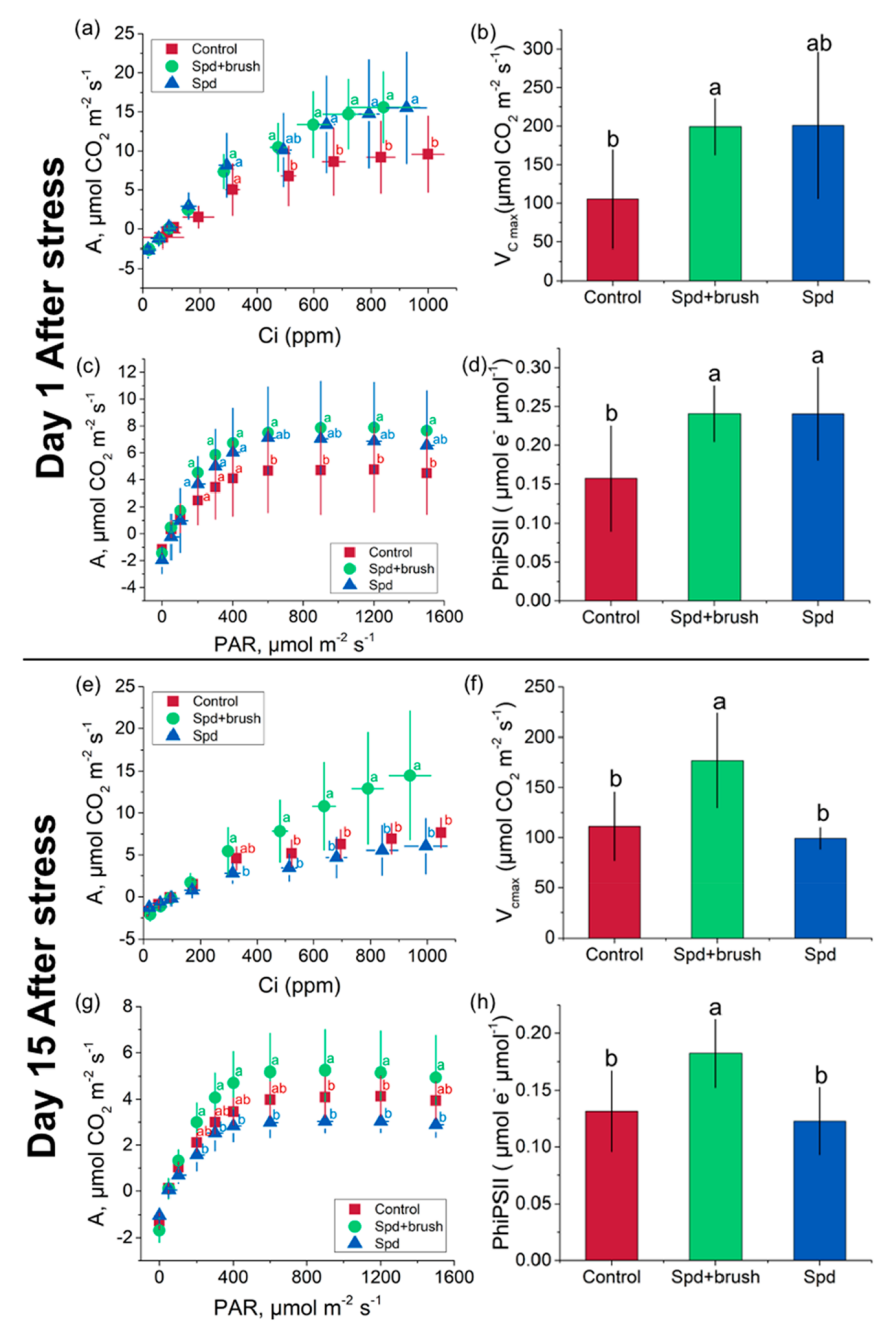
Spd-loaded P[BiBEM-*g*-(PAA_50_-*b*-PNIPAm_50_)]_320_ (SBB50) polymer bottlebrushes enhanced photosynthesis in tomato plants under combined heat (40 °C) and light (2000 μmol m^–2^ s^–1^ PAR) stress for 1.5 h. (a) Carbon response (A-Ci) curve, (b) maximum carboxylation rate V*c*_max_ determined from fitting the data of the A-Ci curve for Ci< 300 ppm, (c) light response (A-PAR) curve, and (d) photosystem II quantum yields of tomato plants treated with either Spd-loaded bottlebrush, free Spd, or Milli-Q water (control) applied with 0.1 vol % Silwet L-77 spreading agent 24 h after bottlebrush treatments. (e) Carbon response (A-Ci) curve, (f) maximum carboxylation rate V*c*_max_ determined from fitting the data of the A-Ci curve for Ci < 300 ppm, (g) light response (A-PAR) curve, and (h) photosystem II quantum yield of tomato plants 15 days after treatment with the Spd-loaded SBB50 polymer bottlebrushes. Letters indicate differences based on an ANOVA test followed by a Fisher’s LSD test for multiple comparisons, *P* ≤ 0.05. Error bars represent standard deviations from five to six replicates. Other photosynthetic parameters are shown in Figure S7.

Free Spd applied at the same concentration as Spd loaded in a bottlebrush resulted in a 44% decrease in the Rubisco carboxylation rate and a drop in the carbon assimilation rate (Figure S5a, b) compared with the control plants, suggesting that foliar application of high concentration (1.24 mM) free Spd decreased photosynthesis by damaging the photosynthetic organelles.^57^ While a low concentration of Spd can help to recover plant photosynthesis under stress, high concentrations of Spd can lead to a decline of the plant maximum photochemical efficiency.^58^ A previous study also suggests that foliar application of 1 mM Spd under light can bleach chlorophyll.^57^ Therefore, we decreased the dosage of free Spd to 0.2 mM to minimize the negative impact from overdosing. Compared to the pure water control, both the Spd-loaded polymer bottlebrush and free Spd treatment increased tomato plant photosynthetic activities under heat and light stress 24 h after application (Figure 3a–d). The Spd-loaded polymer bottlebrush increased carbon assimilation by 63% (Figure 3a) and *V*_*C*max_ by 89% (Figure 3b) and increased the PhiPSII by 53% (Figure 3d) (*P* ≤ 0.05). The Spd delivered via the bottlebrush polymer enhanced photosynthesis by promoting both the Rubisco carboxylation process indicated by higher *V*_*C*max_ (Figure 3b) and the ribulose-1,5-bisphosphate (RuBP) regeneration indicated by a higher carbon assimilation rate at high CO_2_ concentration (Ci ∼ 500–1000 ppm) in a carbon reaction (Figure 3a).^43^ For the light reaction, the Spd bottlebrush treatment increased photon use efficiency of photosystem II, reflected by a higher PhiPSII compared to the control (Figure 3d). Treatment with 0.2 mM free Spd offered similar enhancement in plant photosynthesis under heat and light stress 24 h after application (Figure 3a–d).

To test the ability of the polymer bottlebrushes to provide long-term protection against stress, the plants were stressed again with high heat and excess light 15 days after initial Spd and bottlebrush treatments. The Spd-loaded polymer bottlebrush still promoted photosynthesis of treated leaves under stress conditions, with an 89% increase in carbon assimilation (Figure 3e), a 26% increase in *V*_*C*max_ (Figure 3f), and a 39% increase in PhiPSII (Figure 3h) (*P* ≤ 0.05) compared to control plants, while the photosynthetic activity of the free Spd-treated plants are close to control plants after 15 days (Figure 3 e–h), indicating that the initially applied free Spd is no longer effective. The bottlebrush-only treatment did not significantly impact plant photosynthesis beneficially or negatively (Figure S5a–f), suggesting 0.5 g L^–1^ bottlebrush treatment was not toxic to plants. The temperature-programmed release of Spd delivered by polymer bottlebrushes can protect plants against heat and light stress over a time scale relevant for crop protection (over 2 weeks) and mitigates the negative impacts from Spd overdosing. Spd can effectively mitigate plant ROS stress by upregulating antioxidant enzyme activity. However, free spermidine can be metabolized in plants within 6–10 h and lose their functions as antioxidants.^59^ The Spd loaded into polymer bottlebrushes are only released under stressed conditions, allowing Spd to remain in plants for a longer period of time and provide prolonged plant stress protection. Overall, the SBB50 provided a balance between long-term protection against heat stress and overdosing, providing a desirable therapeutic window of several weeks.

### Polymer Bottlebrush Uptake and Distribution in Tomato Leaves

**Figure 4:**
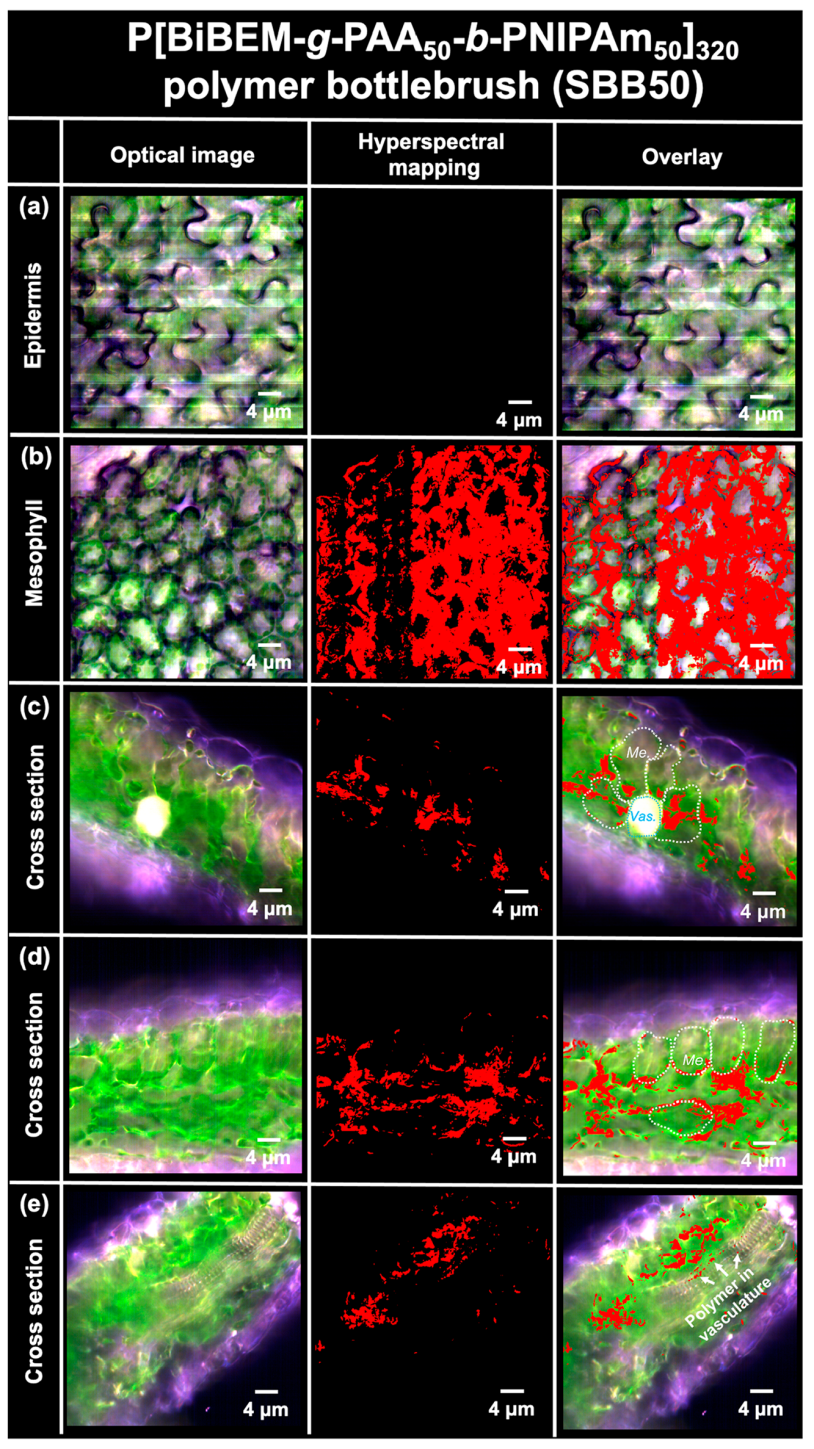
Interactions of CV-loaded P[BiBEM-*g*-(PAA_50_-*b*-PNIPAm_50_)]_320_ (SBB50) bottlebrushes with tomato leaves applied with Silwet L-77 surfactant (0.1 vol %) assessed by enhanced dark-field hyperspectral imaging of leaf epidermis, mesophylls, and cross sections. (a) Epidermis and (b) mesophyll layer of polymer-treated tomato plants. (c) Leaf cross-section images near the plant vasculature bundles (bright white circle). (d) Leaf cross section imaged away from the vasculature bundles. (e) Presence of polymers inside an axial cut of the vasculature bundles. Pixels containing the CV-loaded polymers are highlighted in red based on their hyperspectral signatures (Figure S8). *Me*., mesophyll cell; *Vas*., vasculature bundle.

The ability to release agents in phloem in response to heat stress can make plants more resilient to pests under heat stress.^2,60^ We therefore also evaluated the phloem loading and translocation of polymer bottlebrushes in tomato plants. The polymer bottlebrush interaction with plant leaves after foliar application was studied by enhanced dark-field hyperspectral imaging (DF-HSI). Polymer carriers were labeled with CV to track their distribution *in vivo*.^2,61^ CV can bind strongly with PAA-*b*-PNIPAm polymer nanocarriers and would not release from polymers in tomato plants at room temperature according to our previous study.^2^ Solutions of CV-loaded SBB50 bottlebrushes at 0.5 g L^–1^ polymer concentrations were applied as five 10 μL drops on the adaxial (top) surfaces of tomato leaves. These solutions contained 0.1 vol % Silwet L-77, a common agricultural spreading agent.^2,62,63^ The hyperspectral images were acquired at the leaf epidermis layer and the mesophyll 24 h after foliar application and in leaf cross sections 3 h after application to assess the distribution of polymer nanocarriers in leaves. The bottlebrushes were not detected in the epidermis (Figure 4a) but were found in the mesophyll 24 h after foliar exposure (Figure 4b), indicating the bottlebrushes penetrated through the epidermis effectively and were taken up into mesophyll. Inside the mesophyll, the polymer bottlebrushes distributed mainly at the boundaries of mesophyll cells, around the chloroplasts, with some potentially inside the cell protoplast (Figure 4b). The bottlebrushes were found inside plant cells that were close to the vascular bundles (Figure 4c), suggesting polymer symplastic transport while loading into phloem. The polymer bottlebrushes were also found inside the vasculature in some cross sections, indicating phloem loading occurs (Figure 4e). Away from the vasculature, the bottlebrushes were mainly distributed around cell boundaries, suggesting primarily apoplastic transport in these areas (Figure 4d). The spherical star polymers with similar cross-sectional diameters have shown similar uptake and distribution in leaf compartments compared to the bottlebrush (Figure S10), despite their substantially different lengths.^35^ Thus, polymer nanocarrier transport in tomato leaves was not significantly affected by the largest nanocarrier dimension. This is consistent with a previously reported *ex vivo* nanoparticle plant protoplast interaction model, where nanoparticle uptake into extracted protoplasts and chloroplasts was dominated by the smallest nanoparticle dimension.^64,65^ The uptake to polymer nanocarriers into leaf mesophyll was also confirmed by synchrotron X-ray fluorescence mapping of leaf cross sections (Figure S11).

### Polymer Nanocarrier Translocation Away from Leaves to Other Tissues in Tomato Plants

The leaf uptake and phloem loading of polymer nanocarriers were assessed to evaluate their abilities to deliver agents into different plant organs to combat plant vascular disease.^61^ The high aspect ratio bottlebrushes were loaded with Gd^3+^ to track their movements throughout tomato plants after foliar delivery (Table S3). Stabilities of Gd-loaded nanocarriers in leaves were tested in simulated apoplastic fluid.^61^ Less than 4.5% of loaded Gd leached out of nanocarriers in simulated apoplastic fluid (Table S4), and we have previously shown that less than 2% of free Gd^3+^ ions transport in tomato plants after foliar application.^2^ Solutions of Gd^3+^-loaded polymer bottlebrushes were applied to the second true leaf of tomato plants by deposition of four 5 μL drops with a 1 g L^–1^ polymer concentration, containing 0.1 vol % Silwet L-77. The plants were harvested 3 days after foliar exposure, and the Gd^3+^ concentrations in different plant tissues were quantified with inductively couple plasma mass spectrometry (ICP-MS) after acid digestion.^61^ Local Gd^3+^ concentrations were used to calculate the fraction of the total deposited nanocarrier in each tissue, and a total transport fraction was calculated by normalizing the total amount of Gd^3+^ detected in all tissues outside the leaf exposure zone by the total amount of Gd^3+^ detected, including the exposure zone.^2,61^ The translocation behaviors of shorter (∼80 nm) 320-armed SBB50 and SBB150 bottlebrushes and longer (∼300 nm) LBB50 and LBB150 polymer bottlebrushes were examined to assess the effect of aspect ratio on polymer nanocarrier transport in plants.

**Figure 5:**
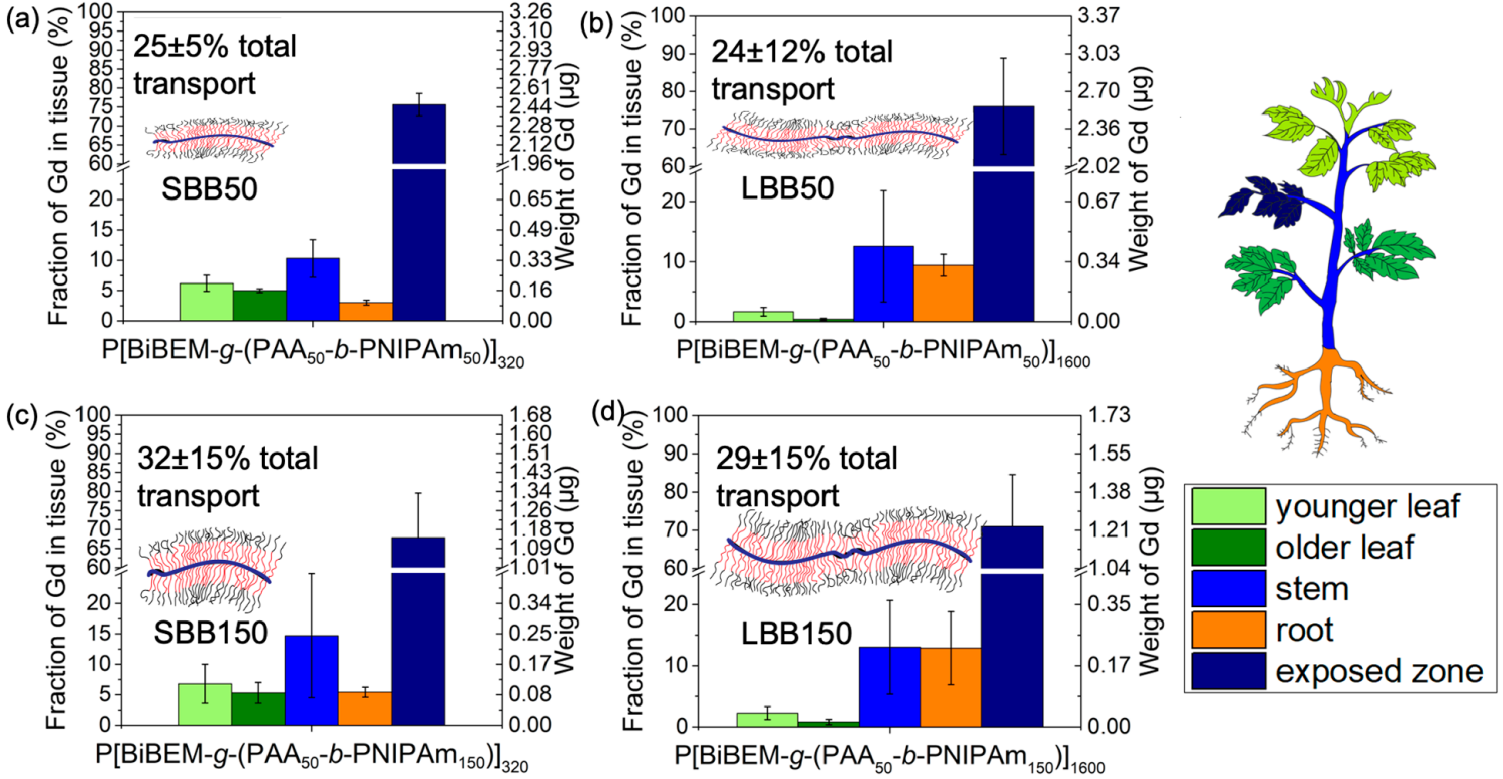
Uptake and transport of Gd^3+^-loaded bottlebrushes in tomato plants after foliar application of 20 μL of a 1 g L^–1^ suspension in 0.1 v/v% Silwet L-77 for (a) P[BiBEM-*g*-(PAA_50_-*b*-PNIPAm_50_)]_320_ (SBB50), (b) P[BiBEM-*g*-(PAA_50_-*b*-PNIPAm_50_)]_1600_ (LBB50), (c) P[BiBEM-*g*-(PAA_50_-*b*-PNIPAm_150_)]_320_ (SBB150), and (d) P[BiBEM-*g*-(PAA_50_-*b*-PNIPAm_150_)]_1600_ (LBB150). Amounts of Gd detected in the different plant tissues are expressed by both fraction of Gd mass applied and total Gd mass in each plant compartment. Error bars represent standard deviations from at least five replicates.

The Gd^3+^ detected in the stem, younger leaves, older leaves, and roots after foliar delivery far exceeded the background Gd^3+^ level in each tissue. (Figure 5). Having a larger aspect ratio and larger size in one dimension did not inhibit nanocarrier uptake and transport on a mass basis, as the total transport fractions of ∼80 nm SBB50 and ∼300 nm LBB50 bottlebrushes were similar to those of the smaller (∼10 nm) PAA_50_-*b*-PNIPAm_50_ star polymers reported in our previous study^2^ (Figure 5a, b, Figure S12a, c). The lack of transport inhibition associated with the high aspect ratio also applies for the polymer carriers with larger cross-sectional diameters (longer arms), as the SBB150 and LBB150 polymer bottlebrushes exhibited statistically indistinguishable total transport from that of PAA_50_-*b*-PNIPAm_150_ star polymers^2^ (Figure 5c, d, Figure S12b, d). The translocation of polymer nanocarriers after foliar application is mostly determined by their net charge and size in the smallest dimension.^64^ Our results suggest high aspect ratio polymers with sizes up to ∼300 nm in length can efficiently transport in the plant vasculature.

Although the total transport fractions were fairly similar for bottlebrushes, the different aspect ratios significantly affected the detailed polymer distributions among different plant tissues (see Figure 5 for individual polymer results and Figure S13 for a direct comparison of different nanocarrier distributions). The shorter (∼80 nm) bottlebrushes exhibited greater translocation to younger and older leaves, while the long (∼300 nm) bottlebrushes mainly transported to roots (*P* ≤ 0.05) (Figure S13a,b). The enhanced translocation to roots for larger bottlebrush polymers is consistent with our previous gold nanoparticle^66^ and star polymer^61^ foliar transport studies, where larger-sized materials preferentially moved to the roots over other tissues. Overall, these results suggest the potential of high aspect ratio polymer bottlebrushes to deliver antimicrobial or other active agents into phloem and other plant organs to manage plant vasculature disease under heat stress.

## Conclusions

We have synthesized high aspect ratio and temperature-responsive P[BiBEM-*g*-(PAA-*b*-PNIPAm)] polymer bottlebrushes that can carry plant stress-regulating agents such as Spd and efficiently phloem load antimicrobial agents such as CV. The polymer bottlebrushes exhibited efficient Spd and CV loading and enhanced agent release at 40 °C compared to 20 °C, at pH 4.5 and 7.5 in buffer and at pH 7 in simulated phloem. The heat-activated Spd delivered by polymer bottlebrushes promoted both the photosynthetic carbon reaction (carbon assimilation, *V*_*C*max_) and light reaction (PhiPSII) after heat and light stress for over 15 days, while the free Spd treatment at the same dose inhibited photosynthetic activity (carbon assimilation, *V*_*C*max_). Free Spd dosed at lower concentration protected plant photosynthesis 1 day after treatment, but not at day 15. The Spd delivered with P[BiBEM-*g*-(PAA-*b*-PNIPAm)] polymer bottlebrushes mitigated negative impacts from high concentration free Spd, and the slow and temperature-controlled Spd release protected plants against heat stress for an extended period of time. Foliar uptake imaging studies showed that the polymers were mainly distributed around the boundaries of mesophyll cells, suggesting apoplastic transport in mesophyll. Polymers were found inside the cells around the vasculature cells, suggesting symplastic transport near the vasculature, consistent with the observed phloem loading. The spherical ∼10 nm 21-armed star polymers and 80 nm 320-armed and 300 nm 1600-armed polymer bottlebrushes exhibited similar total transport from the leaf exposure zone into other plant tissues on a mass basis, indicating that the larger size in one dimension did not inhibit long-range polymer nanocarrier transport in plants. Bottlebrush size did influence the detailed plant tissue distribution, as the larger bottlebrush showed preferential transport to roots over other parts of the plant, consistent with our previous metal nanoparticle transport studies.^66^

Overall, the P[BiBEM-*g*-(PAA-*b*-PNIPAm)] polymer bottlebrushes demonstrate the ability of high aspect ratio (length >50 nm in one dimension) polymer nanocarriers to transport and deliver protective agents into crop plants in a sustained and temperature-responsive manor. The accumulation of longer polymer bottlebrushes in the plant stem and roots potentially enables more effective plant vascular and root disease treatments. These findings can be leveraged to promote sustainability of agriculture by developing new heat-activated approaches that manage plant stress and protect crop yield for longer times with lower frequency of treatments, improving the resilience of agriculture to climate change-induced heat stress. However, the negatively charged polymer bottlebrushes can only carry positively charged agents, while a wide range of agents, including DNA, RNA, and plant phytohormones such as abscisic acid, auxin, and salicylic acid, are negatively charged. Agent carriers with abilities to deliver these agents into mature plants are desired. A similar design strategy to that used here, but with a weak polycation block instead of the weak polyacid block, should be considered for this purpose in the future. The current temperature-responsive polymer bottlebrushes are made with synthetic polymers that are biocompatible but are poorly biodegradable, which limit their sustainability.^31^ Future work can leverage the nanocarrier design rules learned here and develop more sustainable agrochemical carriers with more biodegradable biomaterials such as stimuli-responsive peptides,^67,68^ proteins,^14^ sophorolipids,^69^ and polysaccharides.^70^ Better understanding of the translocation of polymer nanocarriers to the edible parts of crop plants, such as the fruits or grains, is also needed to assess the potential for exposure to these materials through diet. Here, we assessed the uptake of the polymer nanocarriers in tomato plants (a dicot) with particular leaf properties such as stomatal density, cuticle thickness, and trichome density. Future work should also assess the uptake and translocation of the nanocarriers in other crop plants susceptible to heat stress such as wheat, rice, and corn plants (all monocots), considering that their different leaf anatomy and vascular structures may affect the nanocarrier uptake and translocation behaviors. Overall, this temperature responsive agrochemical delivery platform offers growers a new tool for managing climate change-induced stresses. The temperature-actuated release gives growers a large operating window (at least 15 days) to apply treatments that can prevent or lower crop losses from extreme heat events, and that ultimately improves the sustainability of agriculture under climate change.

## Supporting Information Available

Synthesis procedure of polymer bottlebrushes, figures and tables showing ^1^H NMR spectra of P*t*BA-*b*-PNIPAm star polymers and P[BiBEM-*g*-(P*t*BA-*b*-PNIPAm)] bottlebrushes, GPC trace and *M*_n_ of P*t*BA polymer bottlebrushes, DLS size distribution, chemical composition of simulated phloem, images used for build-up spectral library, spectral library for hyperspectral mapping, leaf cross-section images showing polymer transport in vasculature, Gd loading in polymers, total translocation of polymers, polymer distribution in different plant organs, translocation of polymers with the same number concentration and photosynthetic parameters of tomato plants before heat and light stress (PDF)
