# Supplementary material for: Temperature-Responsive Bottlebrush Polymers Deliver a Stress-Regulating Agent In Vivo for Prolonged Plant Heat Stress Mitigation
Source: ACS Sustain Chem Eng. 2023 Feb 14;11(8):3346–58. doi: 10.1021/acssuschemeng.2c06461 (PMC9976702; doi:10.1021/acssuschemeng.2c06461)
Supplement: Supplementary file 1 — sc2c06461_si_001.pdf [file sc2c06461_si_001.pdf]

Supporting information for:

Temperature-Responsive Bottlebrush Polymers Deliver a Stress-Regulating Agent *In Vivo* for Prolonged Plant Heat Stress Mitigation

Yilin Zhang [a,b], *Liye Fu* [c], *Michael R. Martinez* [c], *Hui Sun* [h], *Valeria Nava* [a,b], *Jiajun Yan* [c], *Kurt Ristroph* [a,b], *Saadyah E. Averick* [f], *Benedetto Marelli* [h], *Juan Pablo Giraldo* [g], *Krzysztof Matyjaszewski* [c], *Robert D. Tilton* [b,d,e*] *and Gregory V. Lowry* [a,b*]

a. Department of Civil and Environmental Engineering, b. Center for Environmental Implications of Nano Technology (CEINT), c. Department of Chemistry, d. Department of Chemical Engineering e. Department of Biomedical Engineering, Carnegie Mellon University, Pittsburgh, Pennsylvania 15213, United States.

f. Neuroscience Institute, Allegheny Health Network, Allegheny General Hospital, Pittsburgh, Pennsylvania 15212, United States.

g. Department of Botany and Plant Sciences, University of California, Riverside, California 92521, United States.

h. Department of Civil and Environmental Engineering, Massachusetts Institute of Technology, Cambridge, Massachusetts 02139, United States.

[*] Corresponding author

Phone: (412) 268-2948; fax: (412) 268-7813; email: glowry@cmu.edu (G.V. Lowry);

Phone: (412) 268-1159; fax: (412) 268-7139; email: tilton@cmu.edu (R.D. Tilton);

Number of Figures: 13
Number of Tables: 5
19 pages

Polymer bottlebrush synthesis

Synthesis of P[BiBEM-*g*-P*t*BA$_{50}$]$_{1600}$ polymer bottlebrush.

Synthesis of the longer polymer bottlebrush followed a similar procedure as the shorter polymer bottlebrush. Briefly, 0.05 g (1 equiv) of PBiBEM$_{1600}$ initiator, 4.05 mL of *t*BA (272000 equiv), 7.44 mg $CuBr_2$ (326.4 equiv), 0.029 mL Me_6TREN (1031.4 equiv) and 3.25 mL DMF and 12.98 mL anisole were mixed and sealed in a 25 mL Schlenk flask with a stir bar. The amount of CuBr used was 9.57 mg (653 equiv). The reaction was stopped at ~30% conversion to yield P[BiBEM-*g*-P*t*BA$_{50}$]$_{1600}$ polymer bottlebrush (**Figure S2, Table S1**).

Synthesis of P[BiBEM-*g*-(P*t*BA$_{50}$-*b*-PNIPAm$_{150}$)]$_{320}$ polymer bottlebrush.

A similar polymer but with a longer PNIPAm arm was synthesized with similar approach. Briefly, 0.08 g of P[BiBEM-*g*-P*t*BA$_{50}$]$_{320}$ polymer bottlebrush (1 equiv), 1.01 g NIPAm (237000 equiv), 0.53 mg $CuBr_2$ (63.2 equiv), 0.002 mL Me_6TREN (190 equiv), 0.46 g NaBr and 44.76 mL DMF were mixed and sealed in a 100 mL Schlenk flask with a stir bar. The flask was deoxygenated by purging with N_2 for 120 min. The amount of Cu^0 used was 0.135 g (0.136 cm^{-1}). The reaction was stopped at ~20% conversion to yield P[BiBEM-*g*-(P*t*BA$_{50}$-*b*-PNIPAm$_{150}$)]$_{320}$ polymer bottlebrush (**Figure S1d**).

Synthesis of P[BiBEM-*g*-(P*t*BA$_{50}$-*b*-PNIPAm$_{50}$)]$_{1600}$ polymer bottlebrush.

Briefly, 0.1 g of P[BiBEM-*g*-P*t*BA$_{50}$]$_{1600}$ polymer bottlebrush (1 equiv), 0.34 g NIPAm (326400 equiv), 0.67 mg $CuBr_2$ (326 equiv), 0.0025 mL Me_6TREN (979 equiv), 0.034 g NaBr and 6.8 mL DMF were mixed and sealed in a 10 mL Schlenk flask with a stir bar. The flask was deoxygenated by purging with N_2 for 20 min. The amount of Cu^0 used was 0.035 g (0.23 cm^{-1}). The reaction was stopped at ~25% conversion to yield P[BiBEM-*g*-(P*t*BA$_{50}$-*b*-PNIPAm$_{50}$)]$_{1600}$ polymer bottlebrush (**Figure S1e**).

Synthesis of P[BiBEM-*g*-(P*t*BA$_{50}$-*b*-PNIPAm$_{150}$)]$_{1600}$ polymer bottlebrush.

Briefly, 0.08 g of P[BiBEM-*g*-P*t*BA$_{50}$]$_{1600}$ polymer bottlebrush (1 equiv), 1.01 g NIPAm (1224000 equiv), 0.53 mg $CuBr_2$ (326 equiv), 0.002 mL Me_6TREN (979 equiv), 0.104 g NaBr and 20.3 mL DMF were mixed and sealed in a 50 mL Schlenk flask with a stir bar. The flask was deoxygenated by purging with N_2 for 60 min. The amount of Cu^0 used was 0.104 g (0.23 cm^{-1}).

The reaction was stopped at ~20% conversion to yield P[BiBEM-*g*-(P*t*BA$_{50}$-*b*-PNIPAm$_{150}$)]$_{1600}$ polymer bottlebrush (**Figure S1f**).

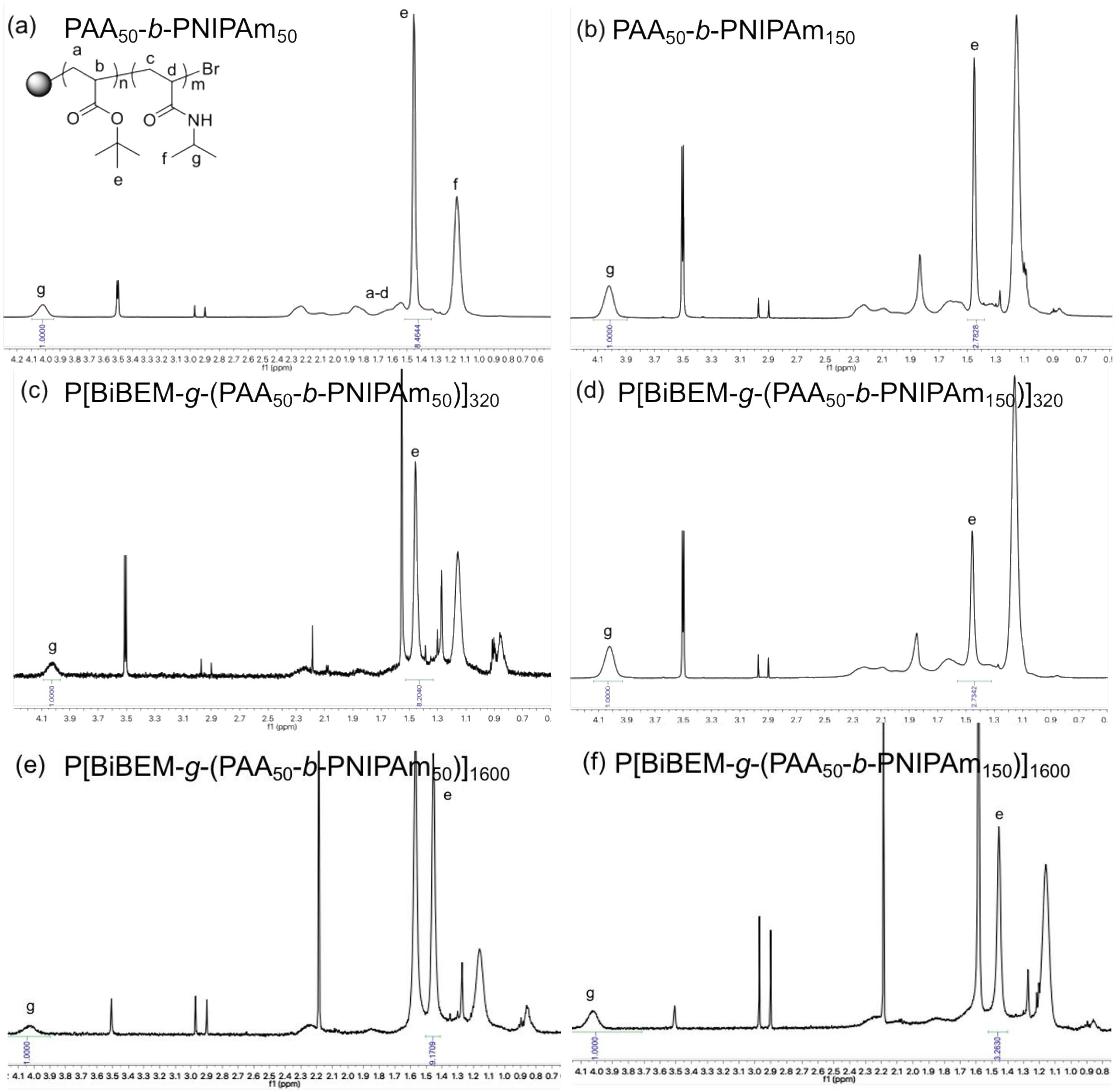

Figure S1. ^{1}H NMR spectra of (a) PAA$_{50}$-*b*-PNIPAm$_{50}$ star polymer, (b) PAA$_{50}$-*b*-PNIPAm$_{150}$ star polymer, (c) P[BiBEM-*g*-(PAA$_{50}$-*b*-PNIPAm$_{50}$)]$_{320}$ bottlebrush, (d) P[BiBEM-*g*-(PAA$_{50}$-*b*-PNIPAm$_{150}$)]$_{320}$ bottlebrush, (e) P[BiBEM-*g*-(PAA$_{50}$-*b*-PNIPAm$_{50}$)]$_{1600}$ bottlebrush and (f) P[BiBEM-*g*-(PAA$_{50}$-*b*-PNIPAm$_{150}$)]$_{1600}$ bottlebrush.

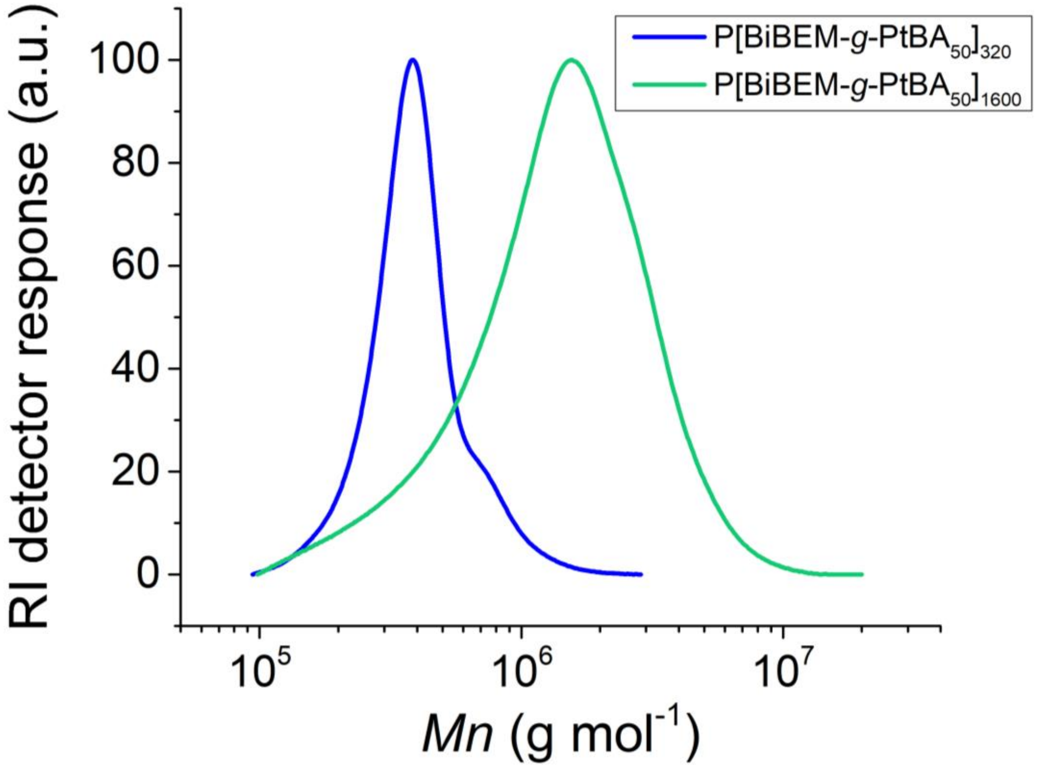

Figure S2. GPC-RI traces of P[BiBEM-*g*-P*t*BA] polymer bottlebrushes with DP=50 in each arm with 320 or 1600 arms in DMF for number average molecular weight (M_n) measurement. The molecular weights measured by MALLS detector are shown in **Table S1**.

Table S1. Number average molecular weights (M_n) of P[BiBEM-*g*-P*t*BA] polymer bottlebrushes in DMF measured by GPC-MALLS.

Sample	***Theoretical* M_n *(g mol^{-1})***	***Measured* M_n *(g mol^{-1})***	***Polydispersity, Đ***
**P[BiBEM-*g*-P*t*BA$_{50}$]$_{320}$**	2.05×10^6	1.89×10^6	1.08
**P[BiBEM-*g*-P*t*BA$_{50}$]$_{1600}$**	1.02×10^7	8.49×10^6	1.06

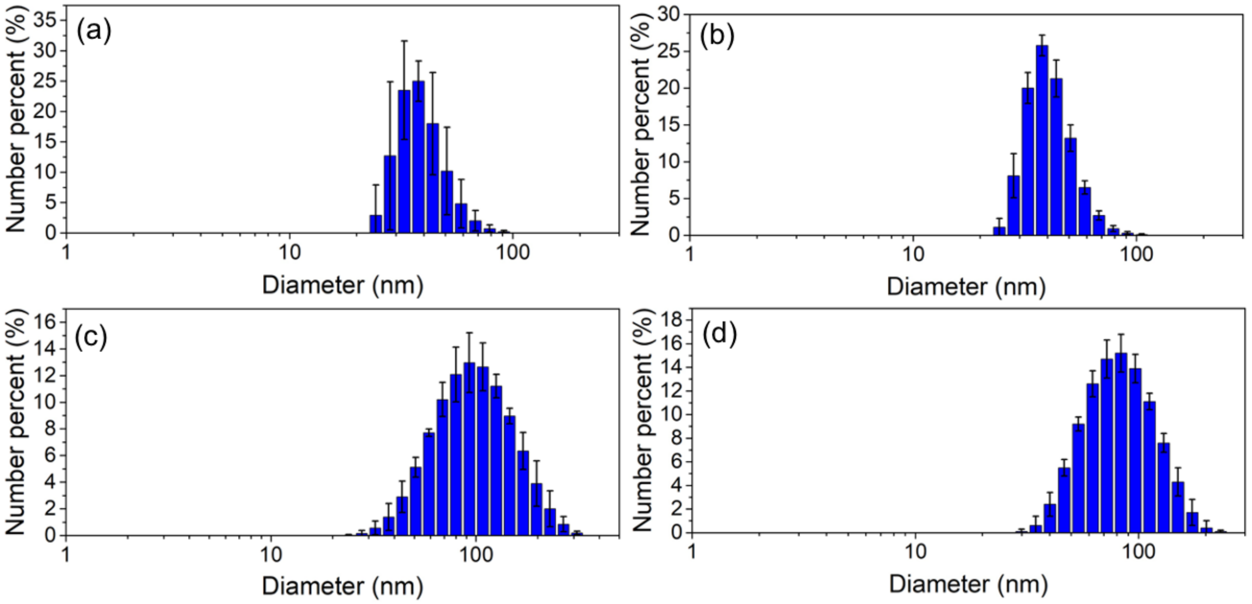

Figure S3. Hydrodynamic diameter distribution of (a) P[BiBEM-*g*-(PAA$_{50}$-*b*-PNIPAm$_{50}$)]$_{320}$ bottlebrush polymers, (b) P[BiBEM-*g*-(PAA$_{50}$-*b*-PNIPAm$_{150}$)]$_{320}$ bottlebrush polymers, (c) P[BiBEM-*g*-(PAA$_{50}$-*b*-PNIPAm$_{50}$)]$_{1600}$ bottlebrush polymers and (d) P[BiBEM-*g*-(PAA$_{50}$-*b*-PNIPAm$_{150}$)]$_{1600}$ bottlebrush polymers measured in 10 mM NaCl water at pH 6.5 at 100 mg L^{-1} polymer concentration.

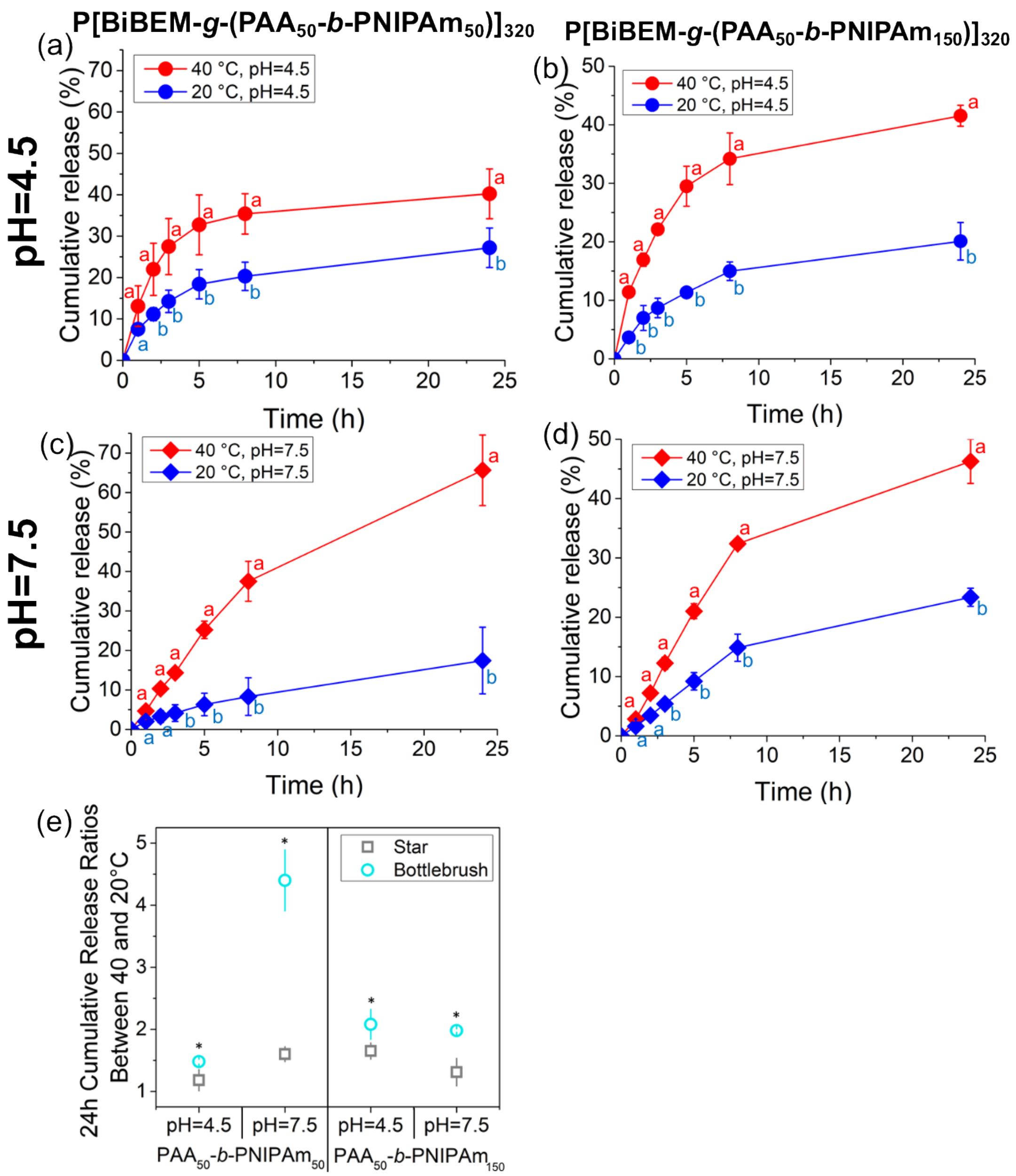

Figure S4. Full CV release profile of 0.5 g L^{-1} P[BiBEM-*g*-(PAA_{50}-*b*-$PNIPAm_{50}$)]$_{320}$ bottlebrush at (a) pH 4.5 and (b) pH 7.5 and P[BiBEM-*g*-(PAA_{50}-*b*-$PNIPAm_{150}$)]$_{320}$ polymer bottlebrushes at (c) pH 4.5 and (d) pH 7.5 in 24 h at 20 °C (blue) and 40 °C (red) in 10 mM phosphate buffer. (e) Ratio of 24 h CV cumulative release for 40 °C and 20 °C for star polymers and 320 armed bottlebrushes. Star polymer release data were reported in our previous publication [1] and are plotted here for comparison. ANOVA test followed by Fisher's Least Significant Difference test for multiple comparisons, $P \leq 0.05$. Error bars represent standard deviations from 3 replicates.

CV release from bottlebrushes in phosphate buffer

The temperature dependent CV release profiles were acquired at plant relevant pH values (4.5 and 7.5) in 10 mM phosphate buffer.[1] Both P[BiBEM-*g*-(PAA_{50}-*b*-$PNIPAm_{50}$)]$_{320}$ and P[BiBEM-*g*-(PAA_{50}-*b*-$PNIPAm_{150}$)]$_{320}$ bottlebrushes exhibited more extensive CV release at 40 °C than at 20 °C. The P[BiBEM-*g*-(PAA_{50}-*b*-$PNIPAm_{50}$)]$_{320}$ bottlebrushes with the smaller PNIPAm block exhibited a greater difference in release for 40 °C vs. 20 °C, at pH 7.5 than at pH 4.5. At pH 7.5, 40 °C incubation resulted in 66±9% cumulative CV release whereas only 17±10% CV released at 20 °C after 24 h (**Figure S4a,b**). At pH 4.5, the P[BiBEM-*g*-(PAA_{50}-*b*-$PNIPAm_{50}$)]$_{320}$ bottlebrushes released 40±6% of loaded CV at 40 °C and 27±5% at 20 °C (**Figure S4a**). This better temperature responsiveness at higher pH is consistent with the temperature and pH responsive behavior of PAA_{50}-*b*-$PNIPAm_{50}$ star polymers with the same arm composition. [1] The P[BiBEM-*g*-(PAA_{50}-*b*-$PNIPAm_{150}$)]$_{320}$ polymer bottlebrushes, with the longer PNIPAm blocks, showed a thermal responsiveness with much weaker dependence on the pH, released 46±4% and 42±2% of CV at 40 °C, at pH 7.5 and 4.5, respectively (**Figure S4c,d**). At 20 °C the CV cumulative release was 23±3% and 20±3% at pH 7.5 and 4.5, respectively (**Figure S4c,d**). Comparing the ratios of 24 h CV cumulative release at 40 and 20 °C, the 320-armed polymer bottlebrushes yielded sharper temperature responsiveness than the 21-armed star polymers with the same PAA to PNIPAm ratio in the arms. (**Figure S4e**) This suggests that the greater arm packing density in cylindrical polymer bottlebrushes (~8 arms nm^{-1}) than the spherical star polymers (~1.5-3 arms nm^{-1}) may allow the PNIPAm shell to more completely occlude the PAA core, enabling sharper temperature control from the PNIPAm block of bottlebrushes.

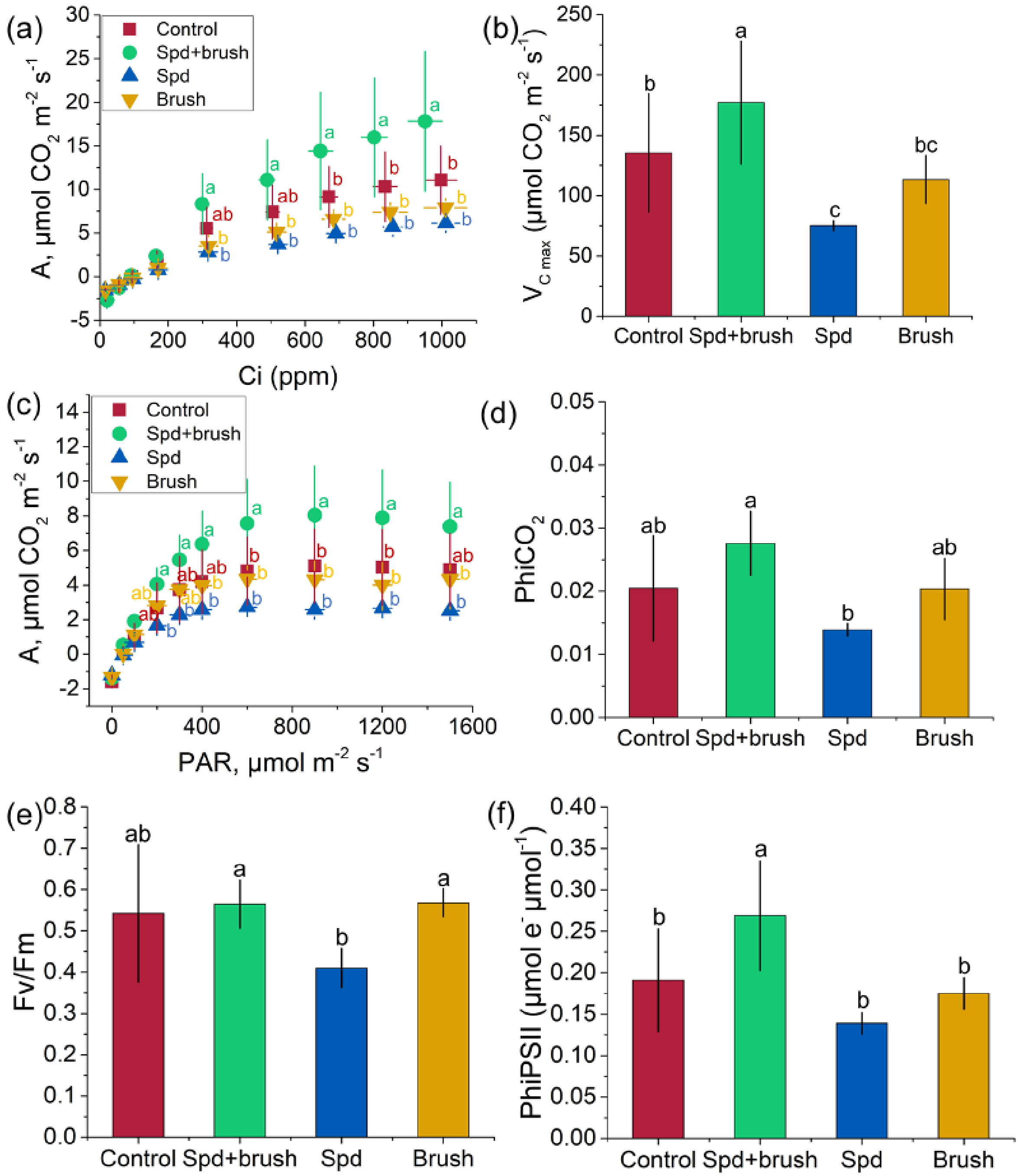

Figure S5. Photosynthetic efficiency of tomato plants 24 h after treatment with Spd-loaded bottlebrush (1.24 mM Spd), bottlebrush, free Spd (1.24 mM) or MilliQ water (control) applied with 0.1 vol% Silwet L-77 under combined heat (40 °C) and light (2000 μmol m^{-2} s^{-1} PAR) stress for 1.5 h. (a) Carbon response (A-Ci) curve (b) maximum carboxylation rate Vc_{max} determined from fits of the data in A-Ci curve for Ci< 300 ppm, (c) light response (A-PAR) curve and (d) CO_2 quantum yield (e) Fv/Fm and (f) photosystem II quantum yield. Letters indicate differences based

on an ANOVA test followed by Fisher's LSD test for multiple comparisons, P ≤ 0.05. Error bars represent standard deviations from 5-6 replicates.

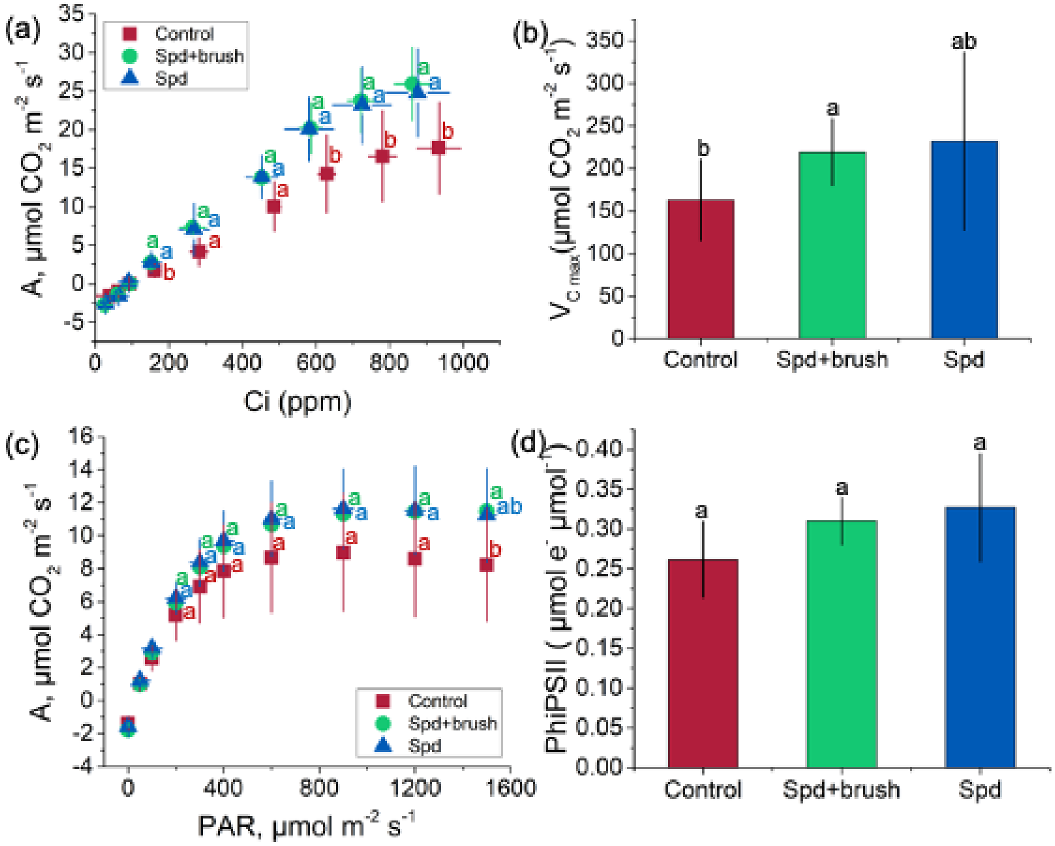

Figure S6. Photosynthetic efficiency of tomato plants 24 h after treatment with Spd-loaded bottlebrush (1.24 mM Spd), bottlebrush, free Spd (0.2 mM) or MilliQ water (control) applied with 0.1 vol% Silwet L-77. (a) Carbon response (A-Ci) curve (b) maximum carboxylation rate Vc_{max} determined from fits of the data in A-Ci curve for Ci< 300 ppm, (c) light response (A-PAR) curve and (d) photosystem II quantum yield. Letters indicate differences based on an ANOVA test followed by Fisher's LSD test for multiple comparisons, P ≤ 0.05. Error bars represent standard deviations from 5-6 replicates.

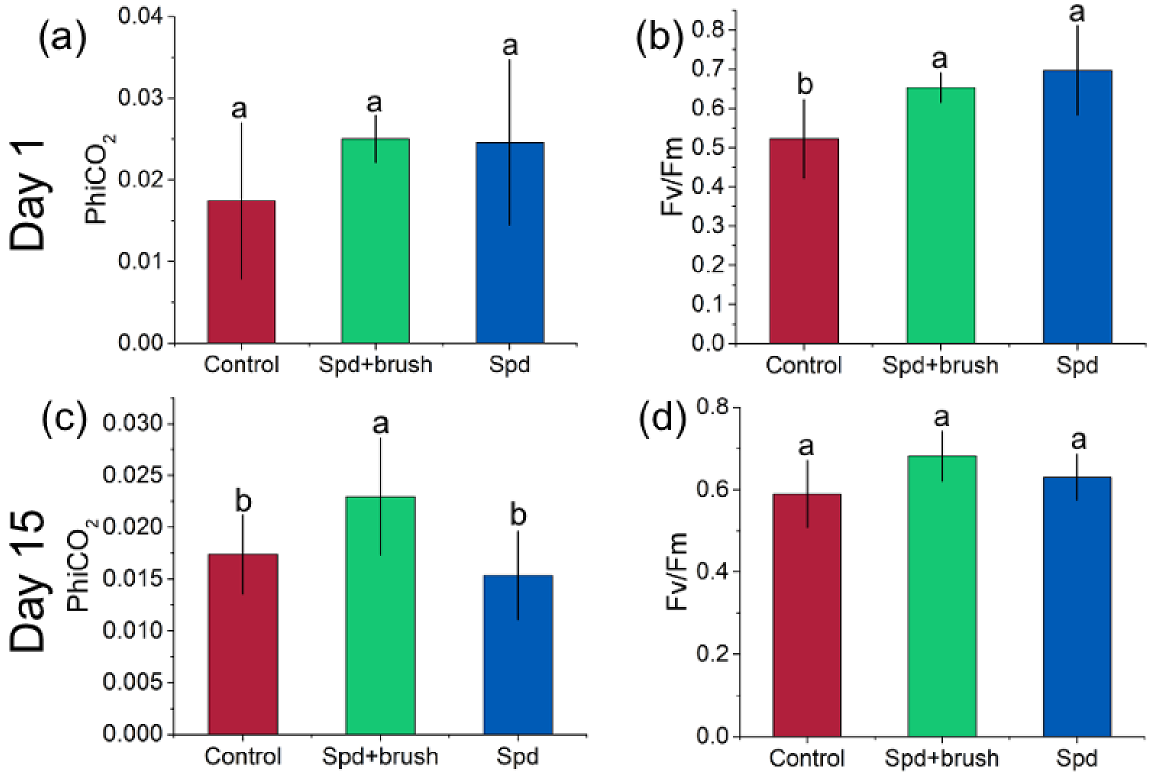

Figure S7. Spd-loaded P[BiBEM-*g*-(PAA_{50}-*b*-$PNIPAm_{50}$)$]_{320}$ polymer bottlebrushes enhanced photosynthesis of tomato plants under combined heat (40 °C) and light (2000 µmol m^{-2} s^{-1} PAR) stress for 1.5 h. (a) CO_2 quantum yield (b) Fv/Fm of tomato plants treated with either Spd-loaded bottlebrush, free Spd or MilliQ water (control) applied with 0.1 vol% Silwet L-77 spreading agent 24h after bottlebrush treatments. (c) CO_2 quantum yield (d) Fv/Fm of tomato plants **15 days after treatments**. Letters indicate differences based on an ANOVA test followed by Fisher's LSD test for multiple comparisons, $P \leq 0.05$. Error bars represent standard deviations from 5-6 replicates.

Table S2. Chemical composition of simulated plant phloem sap at pH 7.0.[2–4]

Chemicals	Concentration (mM)
**Sucrose**	90
**KCl**	15
**Glutamine**	117
**Glutamate**	58.5
**NaCl**	5
**$MgCl_2$**	1
**$CaCl_2$**	1

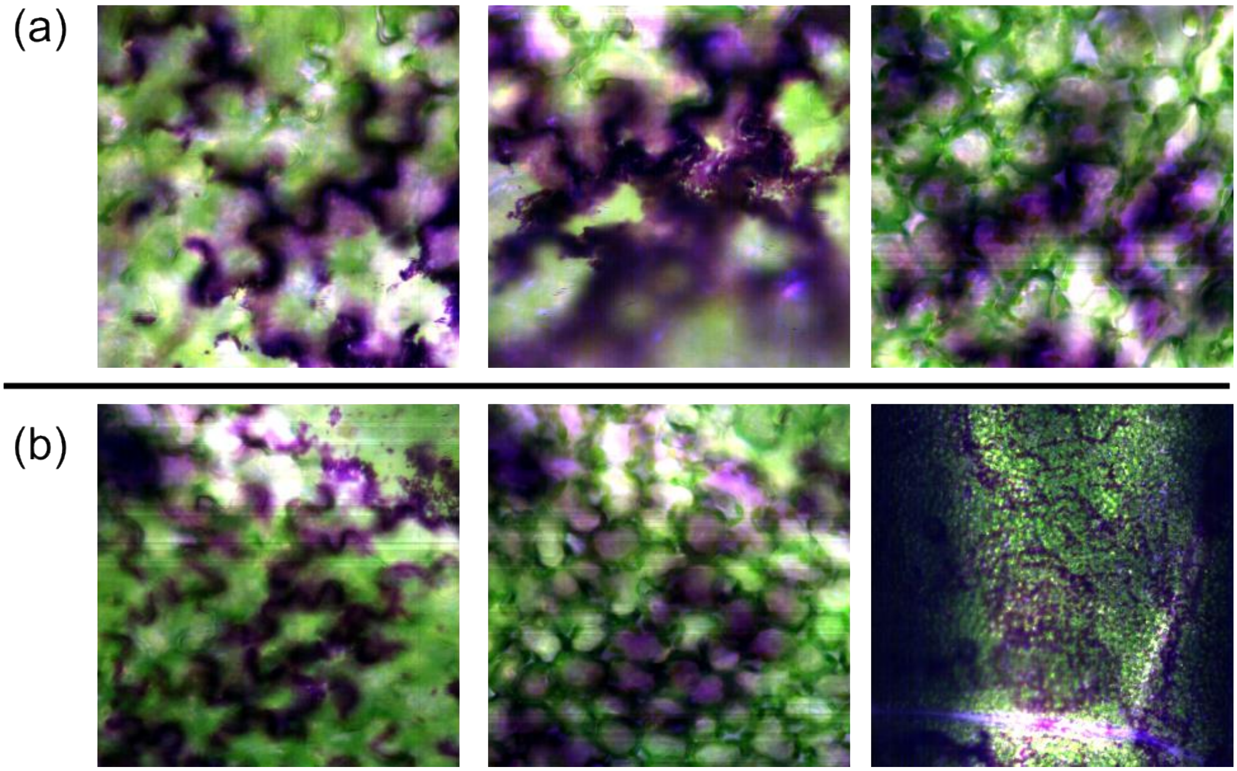

Figure S8. Images used to build the spectral library for CV loaded (a) P[BiBEM-*g*-(PAA$_{50}$-*b*-PNIPAm$_{50}$)]$_{320}$ bottlebrushes and (b) PAA$_{50}$-*b*-PNIPAm$_{50}$ star polymers. The images of CV and CV loaded polymers applied to tomato leaves without Silwet L-77 were used to build the spectral library.

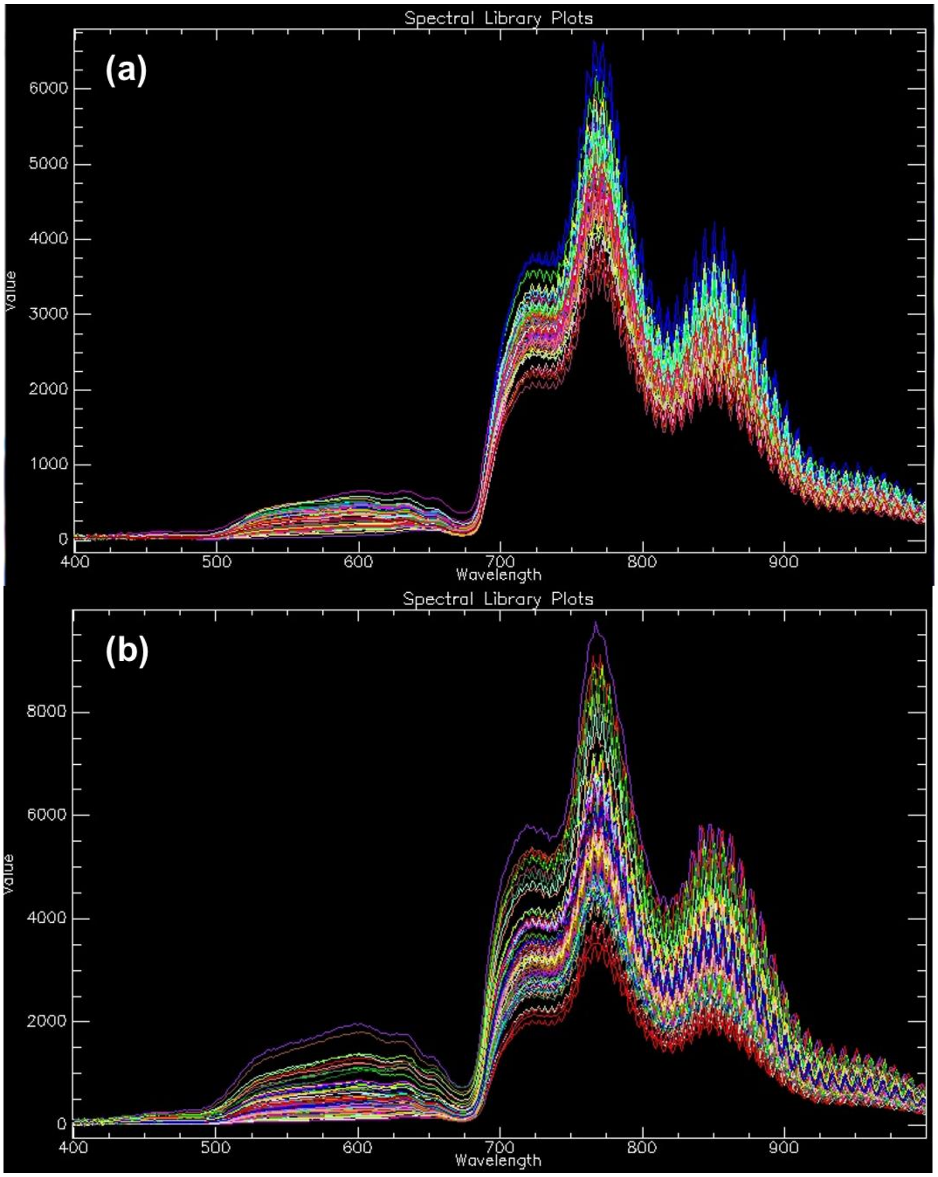

Figure S9. Spectral library of CV loaded (a) P[BiBEM-*g*-(PAA_{50}-*b*-$PNIPAm_{50}$)]$_{320}$ bottlebrushes and (b) PAA_{50}-*b*-$PNIPAm_{50}$ star polymers.

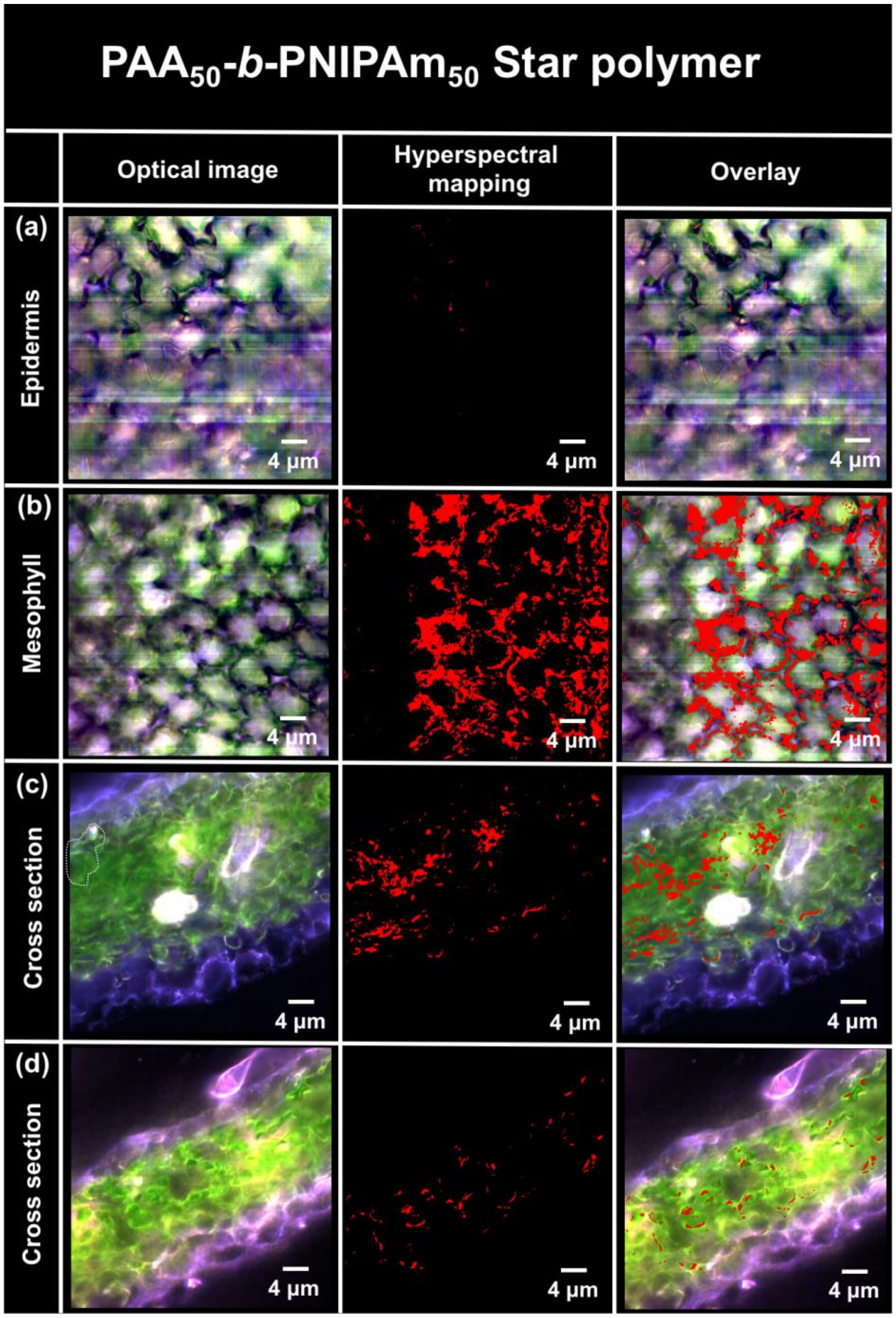

Figure S10. Interactions of CV loaded (PAA$_{50}$-*b*-PNIPAm$_{50}$ star polymer with tomato leaves applied with Silwet L-77 surfactant (0.1 vol%) assessed by enhanced dark field hyperspectral imaging of leaf epidermis, mesophyll and leaf cross sections. The bright white circles in **Figure S7c** are plant vasculature bundles observed in cross-section. **Figure S7d** are imaged at spots away from the vasculature. Pixels containing the CV loaded polymers are highlighted in red based on their hyperspectral signature (**Figure S6**).

a. P[BiBEM-*g*-(PAA_{50}-*b*-$PNIPAm_{50}$)]$_{320}$ polymer bottlebrush

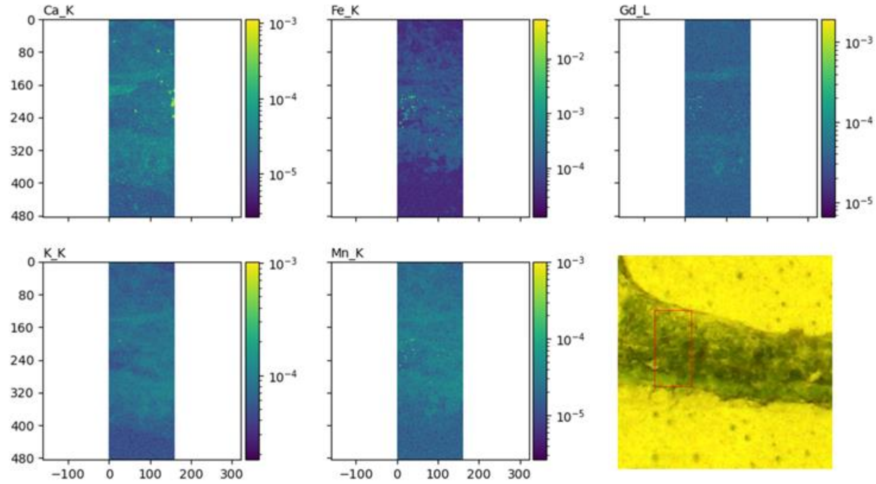

b. P[BiBEM-*g*-(PAA_{50}-*b*-$PNIPAm_{50}$)]$_{1600}$ polymer bottlebrush

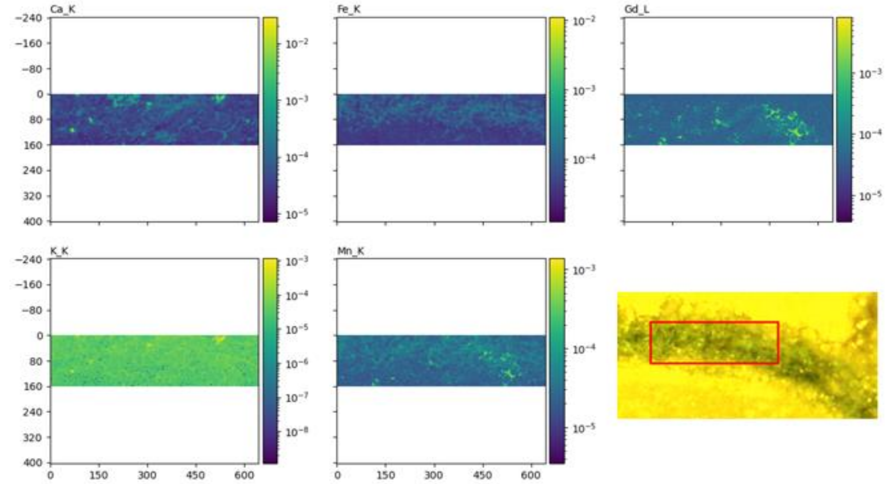

c. PAA_{50}-*b*-$PNIPAm_{50}$ star polymer

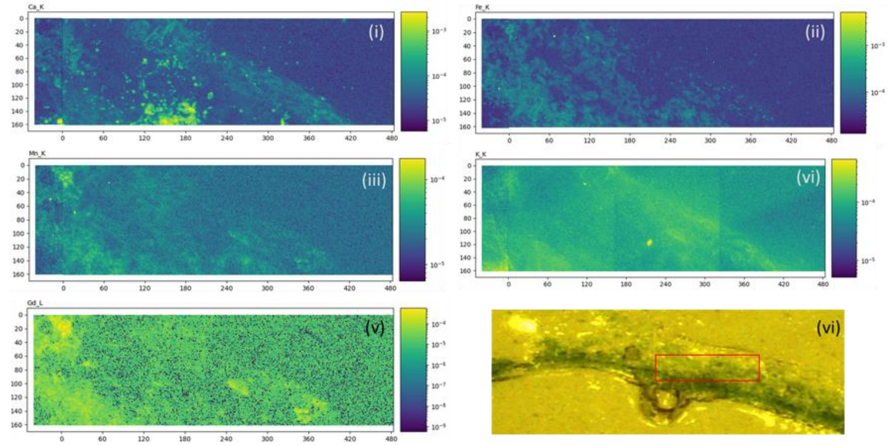

Figure S11. Synchrotron XRF maps collected on 5-ID at NSLSII on freeze dried leaf cross-sections dosed with (a) Gd loaded P[BiBEM-*g*-(PAA_{50}-*b*-$PNIPAm_{50}$)]$_{320}$ polymer bottlebrush, (b)

P[BiBEM-*g*-(PAA_{50}-*b*-$PNIPAm_{50}$)]$_{1600}$ bottlebrush and (c) PAA_{50}-*b*-$PNIPAm_{50}$ star polymer. Color gradients of each element map represents the intensity (log-scale) of the fluorescence emission lines on fitted spectra of (i) Ca K_{α} and K_{β} at 3.7 and 4.0 KeV, respectively (ii) Fe K_{α} and K_{β} at 6.4 and 7.0 KeV, respectively (iii) Mn K_{α} and K_{β} at 5.9 and 6.5 KeV, respectively (iv) K K_{α} and K_{β} at 3.3 and 3.6 KeV, respectively and (v) Gd L_{α} and L_{β} at 6.06 and 6.7, respectively. (vi) Shows an optical image of the approximate scanned area on the freeze-dried cross-section.

Gd loaded nanocarrier distribution in tomato leaf cross section.

Previous work has used Ca, K, Mn, Fe, and K to illustrate plant tissue structures in leaf cross sections.[5] To indicate apoplastic versus symplastic space in plant cross-sections, Ca can be indicative of cell walls since it is a component of the middle lamella of cell walls while K can help to identify the cytoplasm since K is used to help establish cell turgor. [6] Fe and Mn can help to indicate the location of chloroplasts since they are essential elements for photosynthesis.[5] From the XRF mapping we see that at this resolution, it is difficult to distinguish cellular structure in the cross-sections. However, for all the samples, there is high overlap between the location of mapped Gd and mapped Mn and Fe, indicating probable co-localization of the negatively charged polymeric NPs with chloroplasts. For all samples, the fluorescence spectrum at smaller 'hot spot' areas were evaluated to ensure that the signals between Mn, Fe, and Gd were distinguished. Further, there is increased localization of particles closer to vasculature bundles, further suggesting that the polymeric NPs are moving into the plant vasculature.

Table S3. Gd loading in polymer bottlebrushes and star polymers.

**Sample**	***Gd loading in 1g L^{-1} polymer suspension (mg L^{-1})***	***Free Gd out of dialysis bag (mg L^{-1})***
**P[BiBEM-*g*-(PAA_{50}-*b*-$PNIPAm_{50}$)]$_{1600}$**	144.7	0.003
**P[BiBEM-*g*-(PAA_{50}-*b*-$PNIPAm_{150}$)]$_{1600}$**	48.3	0.007
**P[BiBEM-*g*-(PAA_{50}-*b*-$PNIPAm_{50}$)]$_{320}$**	174.7	0.011
**P[BiBEM-*g*-(PAA_{50}-*b*-$PNIPAm_{150}$)]$_{320}$**	95.1	0.015
**PAA_{50}-*b*-$PNIPAm_{50}$**	182.5	0.008
**PAA_{50}-*b*-$PNIPAm_{150}$**	64.9	0.014

Table S4. Concentration of Gd remaining in star polymers and free Gd outside of the star polymer after dialyzing 2 mL of Gd loaded star polymer in 100 mL simulated apoplastic fluid.[7]

**Sample name**	**Gd remain in star polymer suspension (mg L^{-1})**	**Free Gd leached out of star polymers (mg L^{-1})**	**Percent leached (%)**
**PAA_{50}-*b*-$PNIPAm_{50}$**	139.7	0.087	2.4
**PAA_{50}-*b*-$PNIPAm_{150}$**	45.7	0.009	0.7
**P[BiBEM-*g*-(PAA_{50}-*b*-$PNIPAm_{50}$)]$_{320}$**	113.9	0.082	2.8
**P[BiBEM-*g*-(PAA_{50}-*b*-$PNIPAm_{150}$)]$_{320}$**	61.2	0.091	4.6
**P[BiBEM-*g*-(PAA_{50}-*b*-$PNIPAm_{50}$)]$_{1600}$**	141.0	0.022	0.8
**P[BiBEM-*g*-(PAA_{50}-*b*-$PNIPAm_{150}$)]$_{1600}$**	41.4	0.029	3.0

Table S5. Theoretical number average molecular weight (M_n), number average hydrodynamic diameter (D_h), electrophoretic mobility, and apparent zeta potential (ζ) of star polymers.

Sample	M_n[a] (g mol^{-1})	D_h (nm)[b]	Electrophoresis mobility (μm cm V^{-1} s^{-1})[c]	ζ (mV)[d]
**PAA_{50}-*b*-$PNIPAm_{50}$ star**	1.76×10^5	7.8±4.3	-3.80±0.94	-48.5±12.0
**PAA_{50}-*b*-$PNIPAm_{150}$ star**	3.92×10^5	15.5±3.6	-1.54±0.28	-19.7±1.6

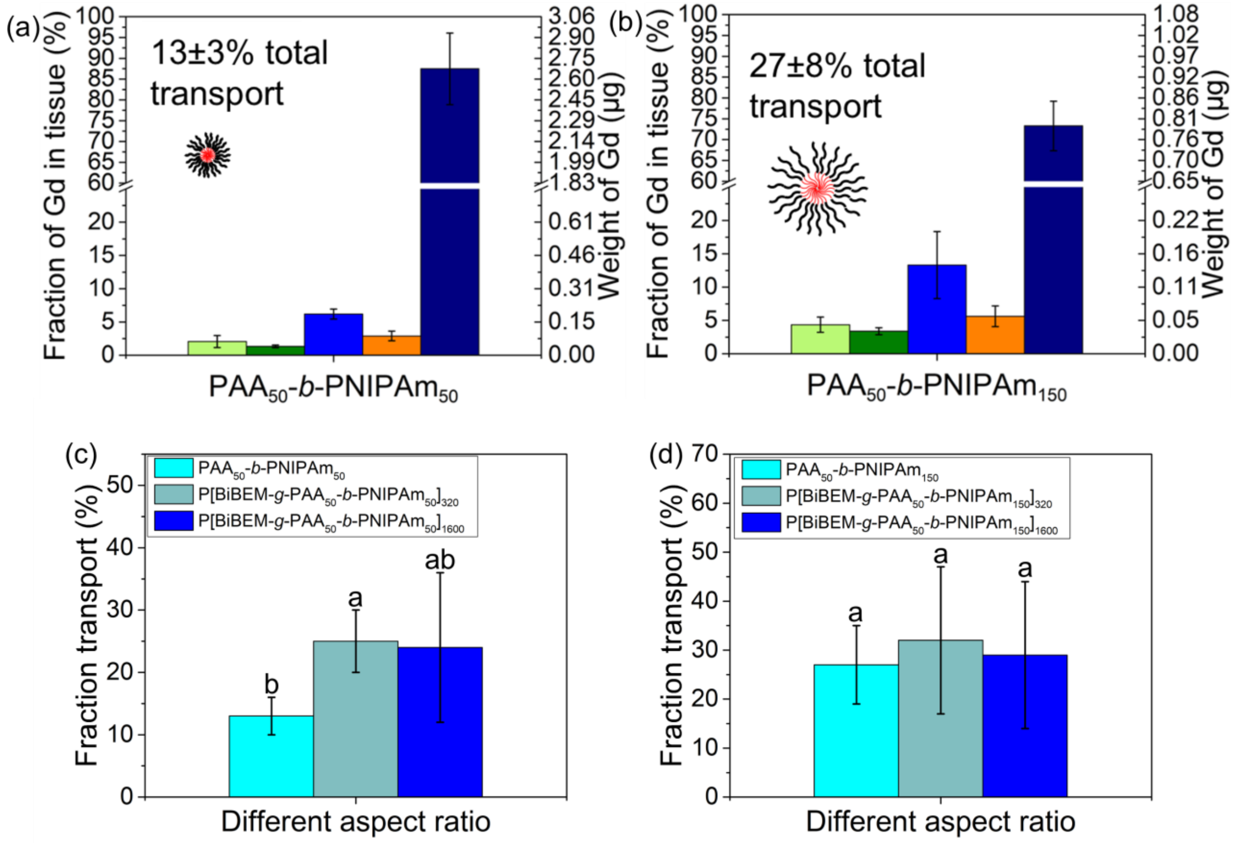

Figure S12. Uptake and transport of Gd^{3+}-loaded star polymers in tomato plants after foliar application of 20 μl of 1 g/L star polymer with 0.1 v/v% Silwet L-77 for (a) PAA_{50}-*b*-$PNIPAm_{50}$ star polymers and (b) PAA_{50}-*b*-$PNIPAm_{150}$ star polymers. Fraction of applied star polymers transported out of the exposed leaf for star polymers and bottlebrushes with (c) PAA_{50}-*b*-$PNIPAm_{50}$ arms and (d) PAA_{50}-*b*-$PNIPAm_{150}$ arms. Error bars represent standard deviations of 5 replicates. ANOVA test followed by Fisher's LSD test for multiple comparisons, $P \leq 0.05$.

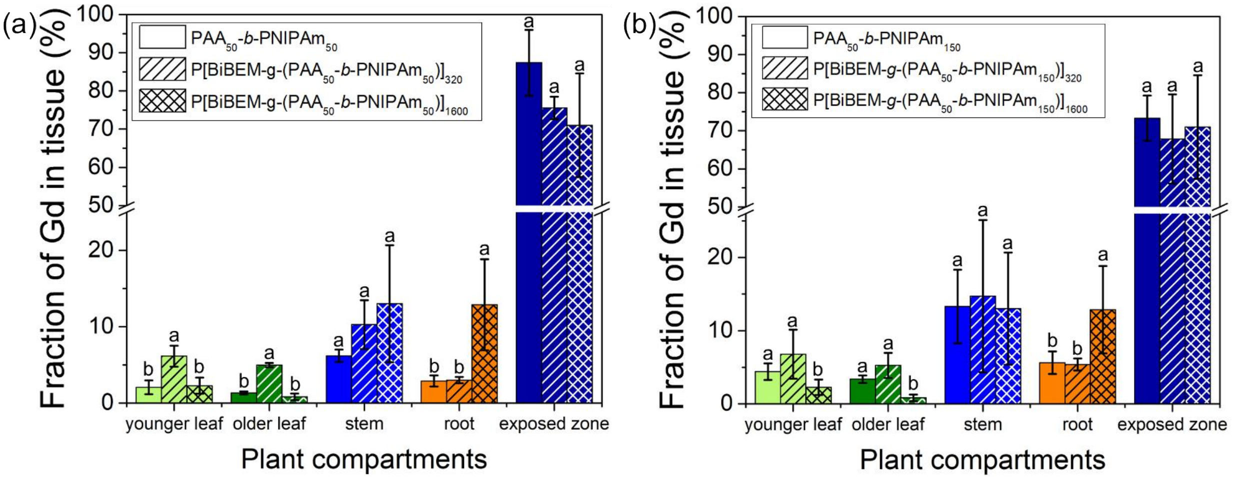

Figure S13. Uptake and transport of Gd loaded bottle brush and star polymers applied onto tomato plant leaves with 0.1 v/v% Silwet L-77 for (a) polymers with PAA_{50}-*b*-$PNIPAm_{50}$ side chains and (b) polymers with PAA_{50}-*b*-$PNIPAm_{150}$ side chains at 1.0 g L^{-1} exposure. Error bars represent standard deviations of 5 replicates. ANOVA test followed by Fisher's LSD test for multiple comparisons, $P \leq 0.05$.

References

(1) Zhang, Y.; Yan, J.; Avellan, A.; Gao, X.; Matyjaszewski, K.; Tilton, R. D.; Lowry, G. V. Temperature- And PH-Responsive Star Polymers as Nanocarriers with Potential for in Vivo Agrochemical Delivery. *ACS Nano* **2020**, *14* (9), 10954–10965. https://doi.org/10.1021/acsnano.0c03140.

(2) Valle, E. M.; Boggio, S. B.; Heldt, H. W. Free Amino Acid Composition of Phloem Sap and Growing Fruit of Lycopersicon Esculentum. *Plant Cell Physiol.* **1998**, *39* (4), 458–461. https://doi.org/10.1093/oxfordjournals.pcp.a029391.

(3) Pérez-Alfocea, F.; Balibrea, M. E.; Alarcón, J. J.; Bolarín, M. C. Composition of Xylem and Phloem Exudates in Relation to the Salt-Tolerance of Domestic and Wild Tomato Species. *J. Plant Physiol.* **2000**, *156* (3), 367–374. https://doi.org/10.1016/s0176-1617(00)80075-9.

(4) Najla, S.; Vercambre, G.; Génard, M. Improvement of the Enhanced Phloem Exudation Technique to Estimate Phloem Concentration and Turgor Pressure in Tomato. *Plant Sci.* **2010**, *179* (4), 316–324. https://doi.org/10.1016/J.PLANTSCI.2010.06.003.

(5) Avellan, A.; Yun, J.; Zhang, Y.; Spielman-Sun, E.; Unrine, J. M.; Thieme, J.; Li, J.;

Lombi, E.; Bland, G.; Lowry, G. V. Nanoparticle Size and Coating Chemistry Control Foliar Uptake Pathways, Translocation, and Leaf-to-Rhizosphere Transport in Wheat. *ACS Nano* **2019**, *13* (5), 5291–5305. https://doi.org/10.1021/acsnano.8b09781.

(6) Taiz, L.; Zeiger, E.; Møller, I. M.; Murphy, A. *Plant Physiology and Development.*; Sinauer Associates Incorporated, 2015.

(7) Zhang, Y.; Fu, L.; Li, S.; Yan, J.; Sun, M.; Pablo Giraldo, J.; Matyjaszewski, K.; D. Tilton, R.; V. Lowry, G. Star Polymer Size, Charge Content, and Hydrophobicity Affect Their Leaf Uptake and Translocation in Plants. *Environ. Sci. & Technol.* **2021**, *55* (15), 10758–10768. https://doi.org/10.1021/acs.est.1c01065.
